# Investigating Pathogenic and Hepatocarcinogenic Mechanisms from Normal Liver to HCC by Constructing Genetic and Epigenetic Networks via Big Genetic and Epigenetic Data Mining and Genome-Wide NGS Data Identification

**DOI:** 10.1155/2018/8635329

**Published:** 2018-09-23

**Authors:** Cheng-Wei Li, Yu-Kai Chiu, Bor-Sen Chen

**Affiliations:** National Tsing Hua University, Kuang-Fu Road, Hsinchu 30013, Taiwan

## Abstract

The prevalence of hepatocellular carcinoma (HCC) is still high worldwide because liver diseases could develop into HCC. Recent reports indicate nonalcoholic fatty liver disease and nonalcoholic steatohepatitis (NAFLD&NASH) and primary biliary cirrhosis and primary sclerosing cholangitis (PBC&PSC) are significant of HCC. Therefore, understanding the cellular mechanisms of the pathogenesis and hepatocarcinogenesis from normal liver cells to HCC through NAFLD&NASH or PBC&PSC is a priority to prevent the progression of liver damage and reduce the risk of further complications. By the genetic and epigenetic data mining and the system identification through next-generation sequencing data and its corresponding DNA methylation profiles of liver cells in normal, NAFLD&NASH, PBC&PSC, and HCC patients, we identified the genome-wide real genetic and epigenetic networks (GENs) of normal, NAFLD&NASH, PBC&PSC, and HCC patients. In order to get valuable insight into these identified genome-wide GENs, we then applied a principal network projection method to extract the corresponding core GENs for normal liver cells, NAFLD&NASH, PBC&PSC, and HCC. By comparing the signal transduction pathways involved in the identified core GENs, we found that the hepatocarcinogenesis through NAFLD&NASH was induced through DNA methylation of *HIST2H2BE*, *HSPB1*, *RPL30*, and *ALDOB* and the regulation of miR-21 and miR-122, and the hepatocarcinogenesis through PBC&PSC was induced through DNA methylation of *RPL23A*, *HIST2H2BE*, *TIMP1*, *IGF2*, *RPL30*, and *ALDOB* and the regulation of miR-29a, miR-21, and miR-122. The genetic and epigenetic changes in the pathogenesis and hepatocarcinogenesis potentially serve as potential diagnostic biomarkers and/or therapeutic targets.

## 1. Introduction

The liver is the largest internal organ of the human body and is involved in many important functions that require high harmonization to control biochemical processes [1]. However, alterations of molecular mechanisms in the liver have been linked to liver diseases such as hepatitis, steatosis, cirrhosis, and hepatocellular carcinoma (HCC). HCC is the end stage of general liver diseases that were classified as nonalcoholic fatty liver disease (NAFLD), autoimmune liver disease, viral hepatitis, alcoholic liver disease, and others. The prevalence of NAFLD, including the more aggressive nonalcoholic steatohepatitis (NASH), is increasing with the growing epidemics of diabetes and obesity. The most frequent symptoms in autoimmune liver diseases (i.e., primary biliary cirrhosis (PBC) and primary sclerosing cholangitis (PSC)), which lead to cholestasis, are fatigue, jaundice, hyperpigmentation, or pruritus. Half a million patients are diagnosed with HCC worldwide each year. It has no significant symptoms in the early stage of HCC through NAFLD&NASH or PBC&PSC and tends to be observed in the advanced stage [2, 3]. Apart from regular surveillance for HCC, understanding the cellular mechanisms of the pathogenesis and hepatocarcinogenesis from normal liver cells to HCC through NAFLD&NASH or PBC&PSC is a priority to slow the progression of liver damage and reduce the risk of further complications.

Recent reports indicate that NAFLD, NASH, PBC, and PSC are responsible for 13%, 4–27%, 3.8%, and 2% HCC cases, respectively. HCC is one of the most deadly malignant tumors, with five-year survival rates ranging from 3% to 28%. Although the reduction of triglycerides in the liver of NAFLD&NASH patients by regular exercise and liver transplantation in PBC&PSC patients can slow the progression of liver damage [4–6], these treatments have some limitations for the complete remedy of liver damage from the perspective of molecular mechanism.

The short noncoding RNA sequences (approximately 21-nucleotide long), microRNAs (miRNAs), were assumed to act by partial repression or degradation of targeted mRNAs. It has been observed that diet-induced obesity in mice results in the differential expression of 6% miRNAs [7]. These changes were similarly observed in human hepatocytes and immortalized liver cell lines exposed to various fatty acids [8]. miRNAs, mostly secreted from cells through active energy-dependent processes via storage in microvesicles, can also circulate freely in the blood [9]. miRNAs were also released by the increased cell death, as it occurs in NAFLD via ballooning degeneration, into the circulation to regulate diverse biological processes including the immune system, cell proliferation, differentiation, and development leading to major advances in the pathomechanism and the hepatogenesis mechanism [10]. Therefore, miRNAs have been proposed as attractive diagnostic biomarkers for investigating, noninvasively, the pathogenesis of NAFLD&NASH (or PBC&PSC) and the carcinogenesis of HCC [11].

Epigenetic regulation, including DNA methylation, histone modification, and chromatin remodelling, results in potentially reversible alterations in gene expression that do not involve permanent changes to the DNA sequence. It has been reported that maternal western diet increases the susceptibility of male offspring to NAFLD [12]. Additionally, by comparing liver biopsies before and after bariatric surgery, it has been observed that weight loss after bariatric surgery leads to NAFLD-associated methylation changes, partially reversible [13]. Therefore, abnormal DNA methylation is the hallmark of the pathogenesis of NAFLD&NASH (or PBC&PSC) and the carcinogenesis of HCC.

DNA methylation, including DNA methylation in the coding region (coding-region methylation) or in the promoter region (promoter-region methylation), regulates the process of mRNA transcription. Promoter region methylation directly affects the binding affinities of miRNAs and transcription factors (TFs) to vary transcriptional profiles [14, 15] while coding region methylation directly attenuates the basal gene expression. The characterization of aberrant DNA methylation is involved in the pathogenesis of NAFLD&NASH (or PBC/PSC) and the carcinogenesis of HCC.

In order to investigate the cellular mechanisms of NAFLD&NASH (or PBC&PSC) pathogenesis and NAFLD&NASH-associated (or PBC&PSC-associated) hepatocarcinogenesis in the liver cells of patients, we constructed the stochastic models of the genome-wide genetic and epigenetic network (GEN) in human cells based on molecular mechanisms, including TF regulations, miRNA repressions, DNA methylation of genes, and protein-protein interactions (PPIs). By applying a system identification method and a system order detection scheme to prune the false positives from the candidate genome-wide GEN constructed by the genetic and epigenetic data mining, we identified the real genome-wide GENs of liver cells in patients with normal liver, NAFLD&NASH, PBC&PSC, and HCC using their corresponding genome-wide mRNA and miRNA expression data and its corresponding DNA methylation profiles. Although the nonsignificant network connections of a liver cell condition have been pruned out from a genome-wide candidate GEN based on a system order detection scheme using the genome-wide expression data, the real genome-wide GEN of a liver cell condition is still complex and complicated owing to the network involving multiple molecular mechanisms. We then applied the principal network projection (PNP) method to the real genome-wide GEN to extract the core network nodes (core proteins/genes/miRNAs), which could constitute signal transduction pathways; i.e., the pathways mediate the intracellular signaling cascade, from the significant network linking energy perspective. By comparing the core pathways with the major differences between normal liver and NAFLD&NASH and between NAFLD&NASH and HCC, we could unravel respectively the cellular mechanisms of NAFLD&NASH pathogenesis and NAFLD&NASH-associated hepatocarcinogenesis. Similarly, by comparing the core pathways with the major differences between normal liver and PBC&PSC and between PBC&PSC and HCC, we could unravel the cellular mechanisms of PBC&PSC pathogenesis and PBC&PSC-associated hepatocarcinogenesis, respectively. We finally proposed network biomarkers, i.e., a set of proteins and miRNAs, as potential diagnostic biomarkers and novel therapeutic drug targets in each liver condition for preventing NAFLD&NASH or PBC&PSC pathogenesis or NAFLD&NASH-associated or PBC&PSC-associated hepatocarcinogenesis. The proposed system biology method also has a potential for use in other liver disease screenings and treatments.

## 2. Results

**Figure 1:**
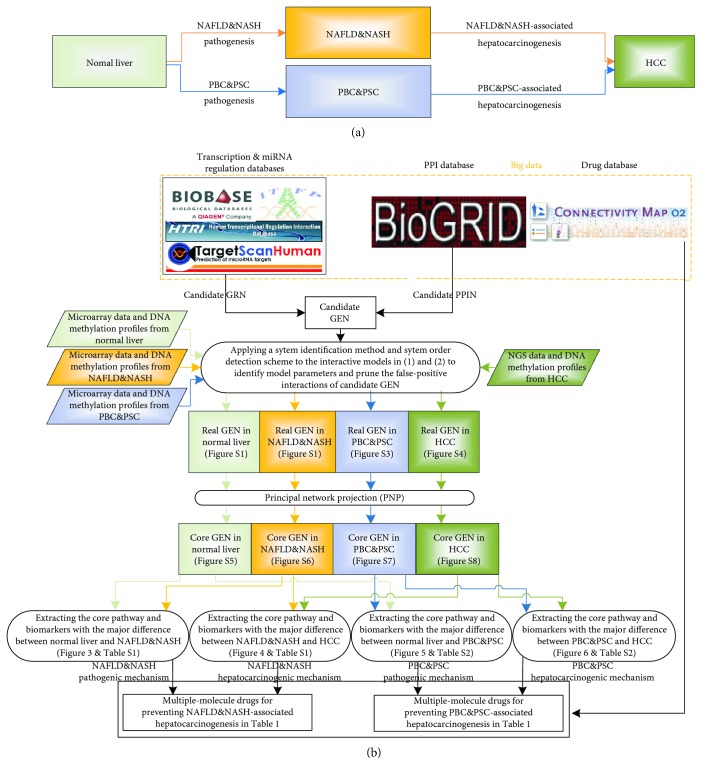
(a) Progression path in pathogenesis and hepatocarcinogenesis. (b) Flowchart for identifying core GENs and pathways.

This study focused on the construction of genome-wide GENs and their core GENs for different liver diseases, to further investigate the hepatocarcinogenic mechanisms, which were then used to design potential drugs for preventing hepatocarcinogenesis in NAFLD&NASH (or PBC&PSC) patients. The hepatocarcinogenic mechanisms for the progression from normal liver cells to HCC were divided into two progression paths with four progression stages, as shown in Figure 1(a). The upper progression path in Figure 1(a) comprises the pathogenesis and hepatocarcinogenesis from normal liver cells to HCC through NAFLD&NASH, represented as NAFLD&NASH pathogenesis and NAFLD&NASH-associated hepatocarcinogenesis, respectively. The lower progression path in Figure 1(a) comprises the pathogenesis and hepatocarcinogenesis from normal liver cells to HCC through PBC&PSC, represented as PBC&PSC pathogenesis and PBC&PSC-associated hepatocarcinogenesis, respectively. Because previous studies have mostly investigated significant proteins or genes instead of using big database mining and genome-wide high-throughput data identification, they may have ignored the effects of the neglected proteins or genes in signaling pathways. In this study, a flowchart for constructing genome-wide GENs, core GENs, and core pathways with network biomarkers for the pathogenesis and hepatocarcinogenesis of HCC is shown in Figure 1(b). First, we constructed the genome-wide candidate GEN in human cells by big genetic and epigenetic database mining. We applied a system identification method and a system order detection scheme to the system models of a genome-wide candidate GEN in human cells to prune the false positives in the candidate GEN and to finally identify the real genome-wide GENs in normal cells, NAFLD&NASH, PBC&PSC, and HCC using genome-wide microarray data and NGS data and their corresponding DNA methylation profiles. Moreover, we applied PNP to the real genome-wide GENs of the four cells to obtain their core GENs in normal cells, NAFLD&NASH, PBC&PSC, or HCC by comparing their core GENs. Furthermore, the signaling pathways of these core GENs were then compared to unravel the hepatocarcinogenic mechanisms from normal liver cells to HCC through NAFLD&NASH or PBC&PSC (see Materials and Methods). The pathogenic and hepatocarcinogenic mechanisms provide valuable insight into the potential drugs for treating patients with liver diseases or HCC.

**Figure 2:**
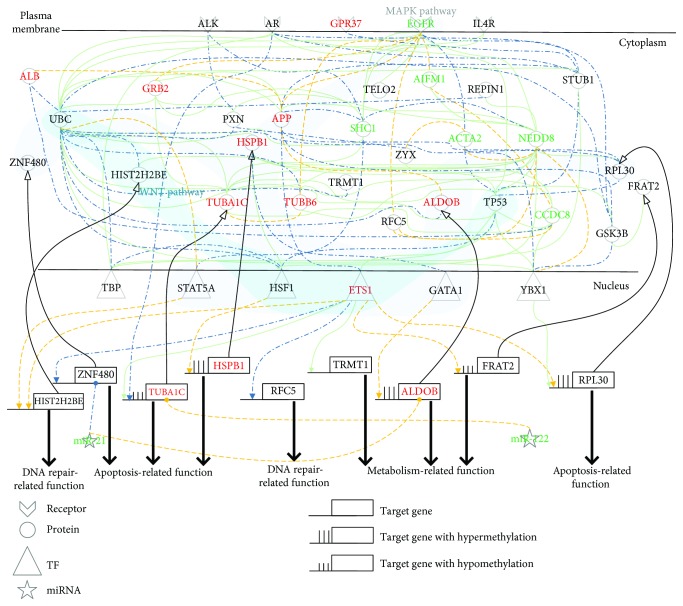
Signaling pathways for investigating cellular mechanisms of progression for pathogenesis from NAFLD&NASH to HCC. The blue dash-dotted lines and the yellow dotted lines represent the specific edges of the real GENs, including PPINs and GRNs, in NAFLD&NASH and HCC, respectively; the green solid lines denote the common edges between NAFLD&NASH and HCC; the red and black symbols indicate specific core members of the real GENs in NAFLD&NASH and HCC, respectively; and the green symbols are the common core members between NAFLD&NASH and HCC. In this figure, these core members are padded to complete the relevant signaling pathways for the convenience of analysis. The cytosolic proteins involved in the MAPK and the WNT pathways were highlighted by light-gray and light-blue bands, respectively. Dysfunctions of both metabolism and apoptosis via DNA hypermethylation and dysregulation of miR-21 contribute to tumorigenesis from NAFLD&NASH to HCC. Dysregulation of miR-21 and miR-122 contributes to tumor invasion and metastasis in HCC.

**Figure 3 fig3:**
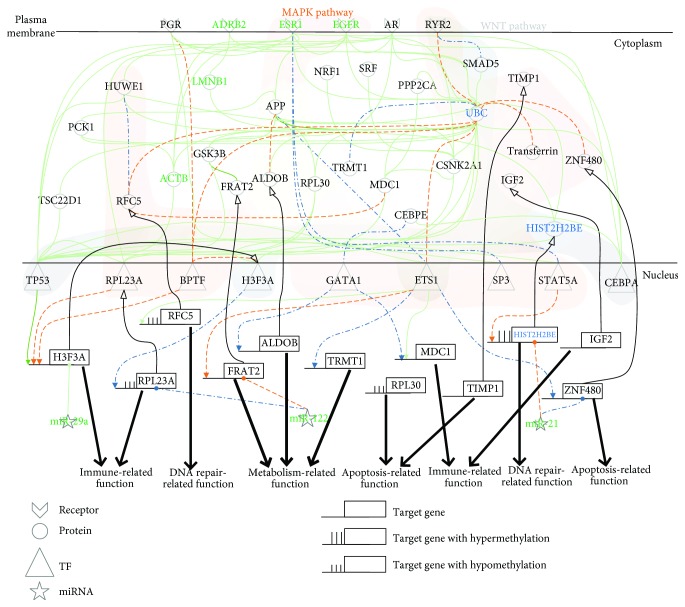
Signaling pathways for investigating cellular mechanisms of progression for pathogenesis from normal to PBC&PSC. The orange dotted lines and the blue dash-dotted lines represent the specific edges of the real GENs, including PPINs and GRNs, in normal and PBC&PSC, respectively; the green solid lines denote the common edges between normal and PBC&PSC; the blue and black symbols indicate specific core members of the real GENs in normal and PBC&PSC, respectively; and the green symbols are the common core members between normal and PBC&PSC. In this figure, these core members are padded to complete the relevant signaling pathways for the convenience of analysis. The cytosolic proteins involved in the MAPK and the WNT pathways were highlighted by light-green and light-blue bands, respectively. Defect of autoimmune response via DNA hypomethylation contributes to the dysfunction of DNA repair and gives rise to PBC&PSC. Dysregulation of miR-21 and miR-122 contributes to the dysfunction of apoptosis and autoimmune in PBC&PSC, respectively.

**Figure 4:**
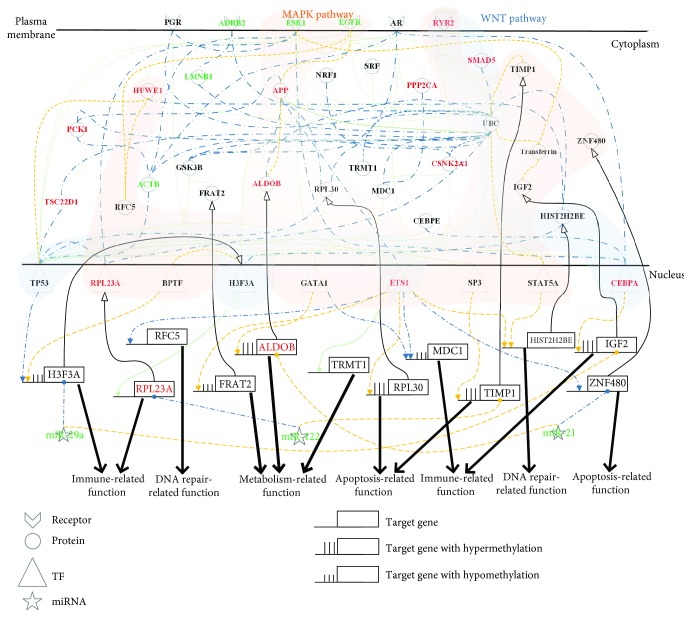
Signaling pathways for investigating cellular mechanisms of progression for pathogenesis from PBC&PSC to HCC. The blue dash-dotted lines and the yellow dotted lines represent the specific edges of the real GENs, including PPINs and GRNs, in PBC&PSC and HCC, respectively; the green solid lines denote the common edges between PBC&PSC and HCC; the red and black symbols indicate specific core members of the real GENs in PBC&PSC and HCC, respectively; and the green symbols are the common core members between PBC&PSC and HCC. In this figure, these core members are padded to complete the relevant signaling pathways for the convenience of analysis. The cytosolic proteins involved in the MAPK and the WNT pathways were highlighted by light-green and light-blue bands, respectively. Dysfunction of metabolism process, apoptosis, and autoimmune via DNA hypermethylation, and dysregulations of miR-21, miR-122, and miR-29a contribute to tumorigenesis from PBC&PSC to HCC. Dysregulation of miR-21, miR-122, and miR-29a contributes to tumor invasion and metastasis in HCC.

We have identified genome-wide GENs for normal liver, NAFLD&NASH, PBC&PSC, and HCC using database mining, microarray data, NGS data, system modeling, and systematic analysis, as shown in Figures S1–S4, respectively (see Materials and Methods). Because the genome-wide real GENs are extremely complicated, we further unraveled the cellular mechanisms of pathogenesis and hepatocarcinogenesis in liver cells from the signal transduction pathway perspective. Therefore, we applied PNP to the genome-wide real GEN to obtain the projection value of each node based on the identified network parameters through the Akaike information criterion (AIC). The top nodes with the highest projection values constitute the core GENs for normal liver, NAFLD&NASH, PBC&PSC, and HCC (Figures S5–S8, respectively), which have complete connections in signal transduction, i.e., whole signaling cascade from receptors to TFs (in Figures 2 [Fig fig3]–4, respectively). Finally, we further clarified the cellular mechanisms of pathogenesis and hepatocarcinogenesis based on the core signaling pathways of each progression stage by making use of the Kyoto Encyclopedia of Genes and Genomes (KEGG) database and the Gene Ontology (GO) database. Based on the core signaling pathways, we also proposed the network biomarkers for a potential drug design to prevent further liver damage of the patients with NAFLD&NASH or PBC&PSC.

### 2.1. Ten-Fold Cross-Validation to Test the Robustness of the Models and AIC

In order to test the robustness of the proposed models in (1) and (2) and AIC in (10), we applied a ten-fold cross-validation test to the mRNA expressions in each cell. We randomly split each data set in normal liver, NAFLD&NASH, PBC&PSC, and HCC into ten sets (e.g., normal liver from 62 patients into 6, 6, 6, 6, 6, 6, 6, 6, 7, and 7 patients). We then repeated the system identification method ten times in normal liver, NAFLD&NASH, PBC&PSC, and HCC. In the protein-protein interaction network (PPIN) of the identified GEN, 88.48% (122,545/138,498) pairs in normal liver, 86.48% pairs (108,357/125,304) in NAFLD&NASH, 86.48% pairs (108,300/125,221) in PBC&PSC, and 14.51% pairs (15,506/106830) in HCC were selected independent of the ten split sets. Additionally, in the gene regulatory network (GRN) of the identified GEN, 19.62% (1212/6178) pairs in normal liver, 18.84% pairs (2298/12,197) in NAFLD&NASH, 19% pairs (2275/11,972) in PBC&PSC, and 33.74% pairs (10,752/31,869) in HCC were selected independent of the ten split sets. Because the low independency of HCC in PPIN results from its largest sample size (369 patients) and a target gene has less candidate TFs (7.3 ± 9.2) in candidate GRN on average as compared to the candidate connecting partners of a protein (15.9 ± 81.9) in candidate PPIN on average, the proposed system identification method can identify a robust real GEN, especially for large sample sizes.

### 2.2. Analysis of Core Pathways to Investigate Underlying Cellular Mechanisms for NAFLD&NASH Pathogenesis and NAFLD&NASH-Associated Hepatocarcinogenesis

**Figure 5:**
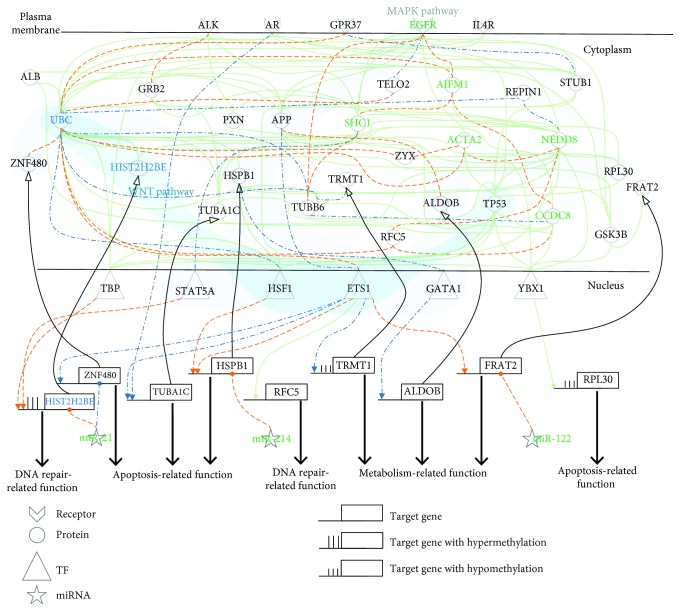
Signaling pathways for investigating cellular mechanisms of progression for pathogenesis from normal to NAFLD&NASH. The orange dotted lines and the blue dash-dotted lines represent the specific edges of the real GENs, including PPINs and GRNs, in normal and NAFLD&NASH, respectively; the green solid lines denote the common edges between normal and NAFLD&NASH; the blue and black symbols indicate specific core members of the real GENs in normal and NAFLD&NASH, respectively; and the green symbols are the common core members between normal and NAFLD&NASH. In this figure, these core members are padded to complete the relevant signaling pathways for the convenience of analysis. The cytosolic proteins involved in the MAPK and the WNT pathways were highlighted by light-gray and light-blue bands, respectively. Dysfunction of DNA repair via DNA hypermethylation and hepatic triglyceride excessive accumulation results in the pathogenesis of NAFLD&NASH. Dysregulation of miR-21 contributes to the dysfunction of apoptosis in NAFLD&NASH.

In the upper progression path of Figure 1(a), the identified core pathways comprise 37 core protein-coding genes, i.e., network biomarkers, involved in at least one core GEN of normal liver cells, NAFLD&NASH, and HCC. The projection values of 37 network biomarkers *D*(*k*) in (17) are also shown in Table S1. We projected these core genes to the KEGG and the GO database mining to get the relevant biological functions and then obtained the core pathways for NAFLD&NASH pathogenesis (Figure 5) and NAFLD&NASH-associated hepatocarcinogenesis (Figure 2).

In the pathogenesis of NAFLD&NASH in Figure 5, we identified eight genes having expression difference between normal liver cells and NAFLD&NASH, i.e., *HIST2H2BE* (*p* value ≤ 1.06 × 10^−1^), *RFC5* (*p* value ≤ 1.5 × 10^−2^), *HSPB1* (*p* value ≤ 6.6 × 10^−2^), *ZNF480* (*p* value ≤ 1.0 × 10^−2^), *TUBA1C* (*p* value ≤ 3.48 × 10^−1^), *RPL30* (*p* value ≤ 1 × 10^−3^), *FRAT2* (*p* value ≤ 1.08 × 10^−1^), and *TRMT1* (*p* value ≤ 5.33 × 10^−1^). Furthermore, three of those genes had basal level differences that might have been caused by DNA methylation on the corresponding genes. According to DNA methylation profiles in the normal liver cells and NAFLD&NASH, we found one gene that had undergone hypermethylation, i.e., *HIST2H2BE* (*p* value ≤ 2.05 × 10^−1^), and two genes that had undergone hypomethylation, i.e., *RPL30* (*p* value ≤ 4.90 × 10^−1^) and *TRMT1* (*p* value ≤ 4.40 × 10^−1^) in a comparison of normal liver cells with NAFLD&NASH cells.

In the hepatocarcinogenesis of NAFLD&NASH in Figure 2, we also identified eight genes with differences in expression between NAFLD&NASH and HCC, i.e., *HIST2H2BE* (*p* value ≤ 1.00 × 10^−3^), *RFC5* (*p* value ≤ 1.00 × 10^−3^), *HSPB1* (*p* value ≤ 1.00 × 10^−3^), *ZNF480* (*p* value ≤ 1.00 × 10^−3^), *TUBA1C* (*p* value ≤ 1.00 × 10^−3^), *RPL30* (*p* value ≤ 1.00 × 10^−3^), *FRAT2* (*p* value ≤ 1.00 × 10^−3^), and *ALDOB* (*p* value ≤ 1.5 × 10^−2^). Furthermore, five of those genes had basal level differences that might have been caused by DNA methylation on the corresponding genes. According to DNA methylation profiles of NAFLD&NASH and HCC, we found three genes that had undergone hypermethylation, i.e., *ALDOB* (*p* value ≤ 8.50 × 10^−1^), *HSPB1* (*p* value ≤ 1.00 × 10^−3^), and *RPL30* (*p* value ≤ 1.00 × 10^−3^), and two genes that had undergone hypomethylation, i.e., *TUBA1C* (*p* value ≤ 1.00 × 10^−3^) and *FRAT2* (*p* value ≤ 1.00 × 10^−3^) in a comparison of NAFLD&NASH with HCC cells.

After extracting two signaling pathways between normal cells and NAFLD&NASH and between NAFLD&NASH and HCC from the core GENs (Figures S5, S6, and S8) as shown in Figures 5 and 2, respectively, we further investigated the cellular mechanisms underlying progression of NAFLD&NASH pathogenesis in Figure 5 and NAFLD&NASH-associated hepatocarcinogenesis in Figure 2. In Figure 5, we observed that in normal liver cells, the insulin receptor (ALK) and the prosaposin receptor (GPR37) were activated to allow ubiquitin C (UBC) to modulate two TFs (ETS1 and TBP) in the WNT and the MAPK signaling pathways to induce DNA repair through the mediation of *HIST2H2BE*, apoptosis through the mediation of *HSPB1*, and metabolism through the mediation of *FRAT2*. To maintain proper DNA repair function, apoptosis and metabolism should be silenced by miR-21, miR-122, and miR-214. For example, miR-21 and miR-214 can silence *HIST2H2BE* and *HSPB1* in normal liver cells without significant liver damage, and miR-122 can silence *FRAT2* to avoid metabolism dysfunction. However, both excessive accumulation of hepatic triglycerides and abnormal DNA repair through the dysregulation of *HIST2H2BE* caused by hypermethylation caused normal liver cells to progress to NAFLD&NASH. In NAFLD&NASH, we observed that oxidative stress could activate epidermal growth factor receptor (EGFR) to facilitate (i) UBC to repress HSF1 in the WNT and MAPK signaling pathways; (ii) APP to activate ETS1 in the MAPK signaling pathway to induce DNA repair function through the mediation of *RFC5*, to activate metabolism through the mediation of *TRMT1*, and to inhibit apoptosis through the mediation of *ZNF480*; and (iii) SHC1 to repress STAT5A in the MAPK signaling pathway. Furthermore, androgen receptor (AR) directly inhibited apoptosis through the mediation of *TUBA1C.* Intriguingly, *HSPB1* caused antiapoptosis without miR-214 silencing and *RFC5* caused DNA repair function. Thus, antiapoptosis and DNA repair could overcome liver damage through the MAPK signaling pathway.

With a series of dysregulations and mutations, NAFLD&NASH developed into HCC through the cellular mechanisms of hepatocarcinogenesis progression, as shown in Figure 2. For example, *ZNF480* silenced by abnormal miR-21 can inhibit apoptosis to facilitate tumorigenesis. In HCC, liver damage and dysregulation due to mutations are still accumulated to facilitate EGFR to repress UBC. Then, UBC modulated STAT5A and HSF1 in the WNT and the MAPK signaling pathways to inhibit DNA repair through the mediation of *HIST2H2BE* and to induce antiapoptosis through the mediation of *HSPB1*, which might finally facilitate tumor growth. Moreover, EGFR also represses TP53 to modulate ETS1 in the MAPK signaling pathway to promote dysfunction of metabolism and apoptosis through the mediation of *FRAT2* and *RPL30.* In addition, the dysregulation of *TUBA1C* caused by abnormal miR-122 silencing and the dysregulation of *ALDOB* caused by both hypermethylation and abnormal miR-21 silencing might facilitate tumor metastasis.

Based on two signaling pathways (Figures 5 and 2) involved in core GENs, we found five network biomarkers, UBC, amyloid precursor protein (APP), SHC-transforming protein 1 (SHC1), EGFR, and V-Ets Avian Erythroblastosis Virus E26 Oncogene Homolog 1 (ETS1), that play a central role in pathogenesis and hepatocarcinogenesis in the upper progression path of Figure 1(a). UBC facilitates a posttranslational modification where ubiquitin is attached to a substrate protein to control intracellular signaling and is involved in protein degradation, DNA repair, cell cycle regulation, kinase modification, endocytosis, and regulation of other cell signaling pathways. Therefore, UBC plays an important role in the appropriate regulation of the human system [16]. APP facilitates posttranslational modifications including glycosylation and phosphorylation. Its primary function is not yet known, although it has been implicated as a regulator of Alzheimer's disease, and may be associated with apoptosis, cell differentiation, and stress responses, and may play a critical role in intracellular signaling [17]. SHC1 has been reported to play an important role in the EGFR signaling pathway [18] and is associated with carcinogenesis and metastasis [19]. It acts as a cell surface protein that binds to epidermal growth factor and a ligand to induce receptor dimerization and tyrosine autophosphorylation, which contributes to cell proliferation and differentiation [20]. Mutations in EGFR are associated with a number of cancers [21], but the precise role of EGFR in HCC is still unknown. ETS1 acts as a transcriptional activator or repressor of numerous genes and is involved in stem cell development, cell death, and tumorigenesis [22]. In addition, we also found nine genes that play a central role in the upper progression path of Figure 1(a), i.e., two DNA repair-related genes (histone cluster 2 (H2be; *HIST2H2BE*) and replication factor C5 (*RFC5*)), four apoptosis-related genes (heat shock 27-kDa protein 1 (*HSPB1*), zinc finger protein 480 (*ZNF480*), tubulin alpha 1c (*TUBA1C*), and ribosomal protein L30 (*RPL30*)), and three metabolism-related genes (aldolase B (*ALDOB*), frequently rearranged in advanced T-cell lymphomas 2 (*FRAT2*), and tRNA methyltransferase 1 (*TRMT1*)).

### 2.3. Analysis of the Signaling Pathways Involved in the Core GENs to Investigate Underlying Cellular Mechanisms for PBC&PSC Pathogenesis and PBC&PSC-Associated Hepatocarcinogenesis

In the lower progression path of Figure 1(a), the identified core pathways comprise 40 core protein-coding genes, i.e., network biomarkers, involved in at least one core GEN of normal liver cells, PBC&PSC, and HCC. The projection values of the 40 network biomarkers *D*(*k*) in (17) are also shown in Table S2. We projected these network biomarkers to KEGG and GO database mining to get the relevant biological functions and then obtained the core pathways for PBC&PSC pathogenesis (Figure 3) and PBC&PSC-associated hepatocarcinogenesis (Figure 4).

In the pathogenesis of PBC&PSC in Figure 3, we identified ten genes with expression differences between normal cells and PBC&PSC, i.e., *HIST2H2BE* (*p* value ≤ 5.62 × 10^−1^), *RFC5* (*p* value ≤ 1.19 × 10^−1^), *TIMP1* (*p* value ≤ 7.5 × 10^−2^), *ZNF480* (*p* value ≤ 1.00 × 10^−3^), *H3F3A* (*p* value ≤ 6.31 × 10^−1^), *ALDOB* (*p* value ≤ 1.55 × 10^−1^), *RPL30* (*p* value ≤ 2.62 × 10^−1^), *FRAT2* (*p* value ≤ 7.16 × 10^−1^), *TRMT1* (*p* value ≤ 1.00 × 10^−3^), *IGF2* (*p* value ≤ 5.44 × 10^−1^), and *MDC1* (*p* value ≤ 1.9 × 10^−2^). Furthermore, four of those genes had basal level differences that might have been caused by DNA methylation of the corresponding genes. With DNA methylation profiles of normal and PBC&PSC for validation, we found one gene that had undergone hypermethylation change, i.e., *HIST2H2BE* (*p* value ≤ 1.2 × 10^−2^), and three genes that had undergone hypomethylation, i.e., *RPL30* (*p* value ≤ 1.00 × 10^−3^), *RPL23A* (*p* value ≤ 1.00 × 10^−3^), and *RFC5* (*p* value ≤ 1.00 × 10^−3^) in a comparison of normal cells with PBC&PSC cells.

In the hepatocarcinogenesis of PBC&PSC in Figure 4, we also identified ten genes having differences in expression between PBC&PSC and HCC, i.e., *HIST2H2BE* (*p* value ≤ 1.00 × 10^−3^), *RFC5* (*p* value ≤ 1.00 × 10^−3^), *TIMP1* (*p* value ≤ 1.00 × 10^−3^), *ZNF480* (*p* value ≤ 1.00 × 10^−3^), *H3F3A* (*p* value ≤ 1.00 × 10^−3^), *RPL30* (*p* value ≤ 1.00 × 10^−3^), *FRAT2* (*p* value ≤ 1.00 × 10^−3^), *ALDOB* (*p* value ≤ 7.2 × 10^−2^), *IGF2* (*p* value ≤ 1.07 × 10^−1^), and *RPL23A* (*p* value ≤ 1.00 × 10^−3^). Furthermore, six of those genes had basal level differences that might have been caused by DNA methylation on the corresponding genes. According to the DNA methylation profiles of PBC&PSC and HCC for validation, we found four genes with hypermethylation, i.e., *ALDOB* (*p* value ≤ 8.30 × 10^−1^), *TIMP1* (*p* value ≤ 1.00 × 10^−3^), *RPL30* (*p* value ≤ 1.00 × 10^−3^), and *IGF2* (*p* value ≤ 1.00 × 10^−3^), and two genes with hypomethylation, i.e., *H3F3A* (*p* value ≤ 1.00 × 10^−3^) and *FRAT2* (*p* value ≤ 1.00 × 10^−3^) in a comparison between PBC&PSC and HCC.

After extracting two signaling pathways between normal cells and PBC&PSC and between PBC&PSC and HCC from the core GENs (Figures S5, S7, and S8) as shown in Figures 3 and 4, respectively, we further investigated the cellular mechanisms underlying the progression of PBC&PSC pathogenesis in Figure 3 and PBC&PSC-associated hepatocarcinogenesis in Figure 4. In Figure 3, we observed that in normal liver cells, ryanodine receptor 2 (RYR2) is activated to facilitate UBC to modulate two TFs (ETS1 and BPTF) in the WNT and MAPK signaling pathways to induce DNA repair through the mediation of *RFC5*, metabolism through the mediation of *FRAT2*, and autoimmune function through the mediation of *MDC1* and *H3F3A*. Proper DNA repair, autoimmune function, and metabolism are maintained by silencing by miR-21, miR-122, and miR-29a. For example, miR-21 and miR-29a can silence *HIST2H2BE* and *H3F3A* in normal liver cells without significant liver damage, and miR-122 can silence *FRAT2* to avoid dysfunctions in metabolism. However, DNA hypomethylation of *RPL23A* contributes to defective autoimmune function causing liver damage, and DNA hypermethylation of *HIST2H2BE* contributes to dysfunction in DNA repair, which might cause normal liver cells to progress to PBC&PSC. In PBC&PSC, we observed that aberrant Ca^2+^ levels could activate RYR2 to facilitate SMAD5 to induce TP53 activated by UBC in the WNT and the MAPK signaling pathways, causing dysregulation of *H3F3A*. EGFR then facilitated APP to repress ETS1 in the MAPK signaling pathway to inhibit both DNA repair through the mediation of *RFC5* and immune function through the mediation of *MDC1*. In addition, ETS1 also induces both metabolism through the mediation of *TRMT1* and apoptosis through the mediation of *ZNF480* (see Figure 3). Furthermore, autoimmune defects through the mediation of *RPL23A* might still be accumulated by both abnormal miR-122 silencing and the dysregulation of H3F3A, and the dysfunction of DNA repair through the dysregulation of *HIST2H2BE* might also still be accumulated without miR-21 silencing, which might facilitate liver damage more seriously through the dysregulations of the MAPK and the WNT signaling pathways.

With a series of dysregulations and mutations, the cellular mechanism for hepatocarcinogenesis progression from PBC&PSC into HCC is shown in Figure 4. For example, *ZNF480* silenced by abnormal miR-21, *RPL23A* silenced by abnormal miR-122, and the dysregulation of *H3F3A* caused by both abnormal miR-29a silencing and hypomethylation might induce the accumulation of autoimmune defects and inhibit apoptosis to facilitate tumorigenesis. In HCC, liver damage and dysregulation due to mutations are still accumulated to facilitate ESR1 that could activate transferrin to repress UBC. Then, UBC could modulate STAT5A and CEBPA in the WNT and the MAPK signaling pathways to inhibit DNA repair through the dysregulation of *HIST2H2BE* and to induce the accumulation of autoimmune defects through the dysregulation of *IGF2*, which might ultimately facilitate tumor growth. In addition, UBC also repressed H3F3A and further suppressed BPTF in the WNT and the MAPK signaling pathways to inhibit *H3F3A* by hypomethylation. Furthermore, EGFR repressed TP53 through the mediations of RFC5 and HUWE1 in the MAPK signaling pathway. Intriguingly, dysregulation of *TIMP1* caused by both abnormal miR-122 silencing and hypermethylation is involved in tumor proliferation. Additionally, the dysregulation of *ALDOB* and *IGF2* caused by hypermethylation and abnormal miRNA regulation through the mediation of miR-21 and miR-29a, respectively, might facilitate tumor metastasis.

Based on two signaling pathways (Figures 3 and 4) involved in core GENs, we found that five network biomarkers, including UBC, APP, EGFR, ETS1, and RYR2, play a central role in pathogenesis and hepatocarcinogenesis in the lower progression path of Figure 1(a). Intriguingly, UBC, EGFR, ETS1, and APP also played an important role in the upper progression path of Figure 1(a). RYR2 is a major mediator of the sarcoplasmic release of stored calcium ions and is involved in metabolism and cell survival [23]. Furthermore, we found seven genes in NAFLD&NASH pathogenesis and NAFLD&NASH-associated hepatocarcinogenesis that also played a central role in PBC&PSC pathogenesis and PBC&PSC-associated hepatocarcinogenesis, i.e., two DNA repair-related genes (*HIST2H2BE* and *RFC5*), two apoptosis-related genes (*ZNF480* and *RPL30*), and three metabolism-related genes (*ALDOB*, *FRAT2*, and *TRMT1*). Moreover, we also identified five genes that play a central role in the lower progression path of Figure 1(a), i.e., one apoptosis-related gene (TIMP metallopeptidase inhibitor 1 (*TIMP1*)), three immune-related genes (ribosomal protein L23a (*RPL23A*), H3 histone family 3A (*H3F3A*), and insulin-like growth factor 2 (*IGF2*)), and one DNA repair-related gene (mediator of DNA-damage checkpoint 1 (*MDC1*)).

### 2.4. Cellular Mechanisms of the Pathogenesis and Hepatocarcinogenesis from Normal Liver Cells to HCC through NAFLD&NASH or PBC&PSC Based on Core Signaling Pathways

Based on the system biology approach shown in Materials and Methods, we constructed the core GENs for the various liver diseases and HCC. We then further investigated the cellular mechanisms of progression from normal liver cells to HCC through the liver diseases by comparing core GENs of the upper or the lower progression paths to extract the core signaling pathways involved in the core GENs for each progression stage in Figure 1(a).

**Figure 6:**
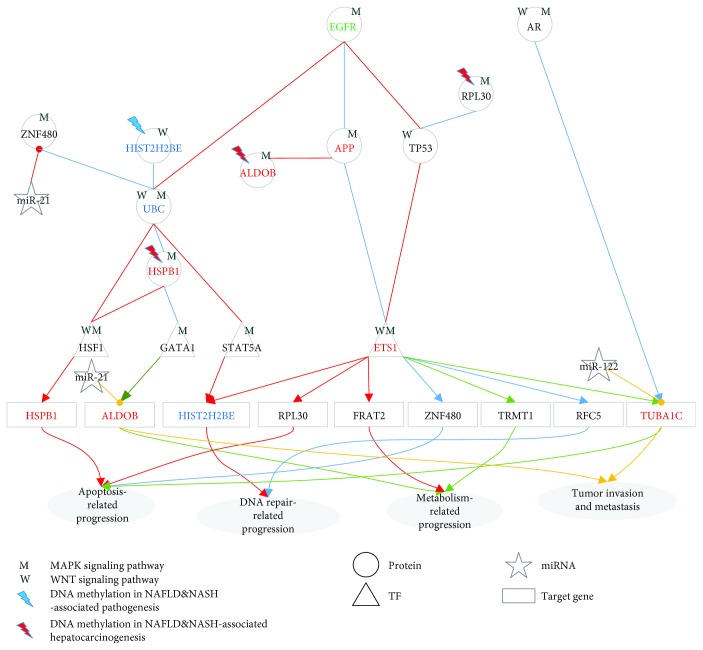
Progression mechanisms for the pathogenesis of NAFLD&NASH and the hepatocarcinogenesis through NAFLD&NASH. The blue, red, green, and yellow lines represent the interactions (or regulations) in NAFLD&NASH pathogenesis, NAFLD&NASH hepatocarcinogenesis, the hepatocarcinogenesis of normal liver through NAFLD&NASH, and the aggressive tumor progression, respectively, based on Figures 5 and 2. The epigenetic modification of HIST2H2BE could facilitate the dysfunction of metabolism-related progression through the dysregulations of the WNT and the MAPK signaling pathways resulting in NAFLD&NASH. The accumulated epigenetic modifications and dysregulations of miR-21 could facilitate the dysfunctions of metabolism-related, apoptosis-related, and DNA repair-related progression through dysregulations of the WNT and the MAPK signaling pathways resulting in HCC. Dysregulation of miR-21 and miR-122 could contribute to tumor invasion and metastasis to facilitate further aggressive tumor progression.

After the investigations of Figures 5 and 2 for NAFLD&NASH pathogenesis and NAFLD&NASH-associated hepatocarcinogenesis as shown in Figure 1(a), it can be seen that the general progression mechanism for the upper progression path is through the WNT and the MAPK signaling pathways, inducing DNA repair, apoptosis, and metabolism. Further, the liver homeostatic balance is maintained by miR-21, which silences the *HIST2H2BE* gene thereby promoting proper cellular functions such as the development, senescence, and proliferation of cells and the development and function of erythroblasts in normal liver cells; miR-214 silences the *HSPB1* gene to avoid unnecessary antiapoptosis, and miR-122 silences the *FRAT2* gene to avoid dysfunction of metabolism causing liver damage. The accumulation of liver damage is caused by steatosis, and *HIST2H2BE* is regulated by hypermethylation through the MAPK signaling pathway, which facilitates abnormal DNA repair and the progression of normal liver cells to NAFLD&NASH, as shown in Figure 5. Furthermore, *ZNF480* can be inhibited and *HSPB1* can be activated to cause antiapoptosis, overcoming liver damage. However, the dysfunction of antiapoptosis caused oncogene proliferation and survival through the dysregulation of *ZNF480* by abnormal miR-21 silencing and through the dysregulation of *HSPB1* by hypermethylation, facilitating the dysregulation of the MAPK signaling pathway. Additionally, the dysfunction of metabolism caused by hypermethylation of *ALDOB* allows NAFLD&NASH to develop into HCC. Interestingly, abnormal miR-21 can still be accumulated to induce the dysregulation of *ALDOB*, and abnormal miR-122 can also be accumulated to induce the dysregulation of *TUBA1C*, which might facilitate tumor metastasis and invasion in HCC, as shown in Figure 2. To integrate the description given in Figures 5 and 2 of the NAFLD&NASH pathogenesis and NAFLD&NASH-associated hepatocarcinogenesis as shown in Figure 1(a) and to consider the impact of both miRNA regulation and DNA methylation on the mechanisms of progression in the upper progression path, we further investigated the role of miRNA regulation and DNA methylation in the mechanisms of progression in the upper progression path. Our objective was to discover the potential diagnostic biomarkers or drug targets that are summarized in Figure 6, with a more detailed description in Discussion.

**Figure 7:**
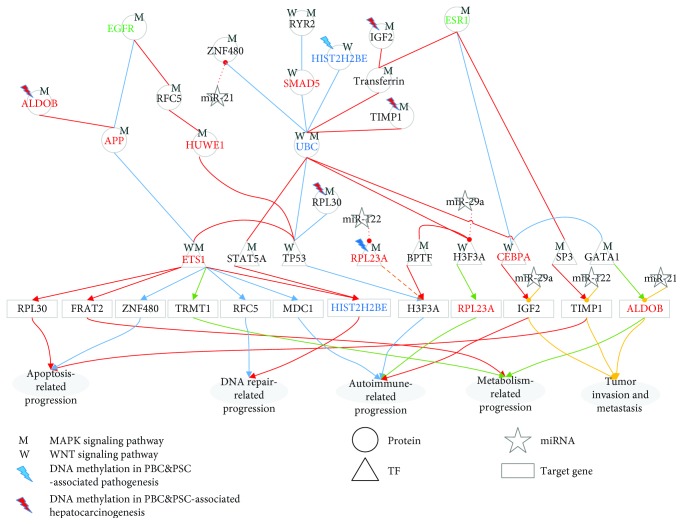
Progression mechanisms for the pathogenesis of PBC&PSC and the hepatocarcinogenesis through PBC&PSC. The blue, red, green, and yellow lines represent the interactions (or regulations) in PBC&PSC pathogenesis, PBC&PSC hepatocarcinogenesis, the hepatocarcinogenesis of normal liver through PBC&PSC, and the aggressive tumor progression, respectively, based on Figures 3 and 4. The epigenetic modifications of HIST2H2BE and RPL23A could facilitate the dysfunction of autoimmune-related progression through dysregulations of the WNT and the MAPK signaling pathways resulting in PBC&PSC. The accumulated epigenetic modifications and the dysregulation of miR-29a, miR-122, and miR-21 could facilitate the dysfunction of metabolism-related, apoptosis-related, autoimmune-related and DNA repair-related progression through the dysregulation of the WNT and the MAPK signaling pathways resulting in HCC. Dysregulation of miR-21, miR-122, and miR-29a could contribute to tumor invasion and metastasis to facilitate further aggressive tumor progression.

We then observed that the general mechanism for the lower progression path is also through the WNT and the MAPK signaling pathways via different epigenetic regulations compared with the upper progression path that induces DNA repair, metabolism, and autoimmune function based on the investigations of Figures 3 and 4 for the PBC&PSC pathogenesis and PBC&PSC-associated hepatocarcinogenesis, as shown in Figure 1(a). To further maintain liver homeostatic balance by miR-29a silencing of *H3F3A* to promote proper gene regulation such as the regulation of *RPL23* to cause autoimmune function in normal liver cells, miR-21 can silence *HIST2H2BE* to avoid unnecessary DNA repair, and miR-122 can silence *FRAT2* to avoid dysfunction of metabolism causing liver damage. In contrast to normal liver cells that progress to NAFLD&NASH, *RPL23A* regulated by hypomethylation caused defects in the autoimmune response to promote more severe liver damage through the WNT and the MAPK signaling pathways, and *HIST2H2BE* was also regulated by hypermethylation to facilitate abnormal DNA repair through the MAPK signaling pathway in the progression of normal liver cells to PBC&PSC, as shown in Figure 3. Therefore, we realized that NAFLD&NASH, as a reversible liver disease, might allow repairing liver damage by an autoimmune response, but the dysfunction of the autoimmune response might cause more severe liver damage in PBC&PSC. Most cellular mechanisms share some similarity to NAFLD&NASH progressing to HCC other than the dysfunction of the autoimmune response caused by the dysregulation of both *H3F3A* and *RPL23A* through abnormal miR-29a and miR-122 silencing, respectively, to facilitate the progression of PBC&PSC into HCC. Furthermore, abnormal miR-122 can still be accumulated to cause the dysfunction of apoptosis through the dysregulation of *TIMP1*, and abnormal miR-29a can still be accumulated to cause the dysfunction of autoimmune through the dysregulation of *IGF2*, and abnormal miR-21 can still be accumulated to cause the dysfunction of metabolism through the dysregulation of *ALDOB*, which might facilitate tumor metastasis and invasion in HCC, as shown in Figure 4. To integrate the descriptions of Figures 3 and 4 of PBC&PSC pathogenesis and PBC&PSC-associated hepatocarcinogenesis as shown in Figure 1(a) and to consider the impact of both miRNA regulation and DNA methylation on the mechanisms of progression in the lower progression path, we further investigated the role of miRNA regulation and DNA methylation in the lower progression path. This provided insight into the development of the potential diagnostic biomarkers or drug targets summarized in Figure 7, with a more detailed description in Discussion.

## 3. Discussion

Recently, there has been an intensive investigation into epigenetic regulation and the role it plays in the progression of liver disease to HCC [24]. Epigenetic dysregulation promotes the pathogenesis of hepatocytes and facilitates the development of HCC [25]. In principle, epigenetic regulations include DNA methylation, histone modification, and miRNA transcription [26]. Histone modifications consist of acetylation, methylation, phosphorylation, and ubiquitination, and although they cannot be represented by an interactive model, they still caused significant variations in the genetic regulatory parameters in our method. Hence, we considered the impact of both DNA methylation and miRNA regulation in GENs on pathogenesis and hepatocarcinogenesis from normal liver cells to HCC through different liver diseases. Because aberrant DNA methylation causes abnormal gene expression, DNA methylation is also regarded as a key process in the pathogenesis and hepatocarcinogenesis from normal liver cells to HCC through liver diseases. Recent studies have indicated that hypermethylation induced gene silencing, inhibit gene expression, and affect genomic stability [27]. miRNAs are small noncoding RNAs that modulate the transcription and translation of target genes and regulate different biological functions in the human liver. In addition, miRNAs are involved in posttranscriptional regulations that are associated with the proliferation, differentiation, and death of cells and with carcinogenesis [28]. Using the proposed system biology approach, we identified core miRNAs and further extracted the core GENs and the involved signaling pathways that could realize cellular mechanisms for the progression of normal liver cells to HCC through liver diseases and could also provide drug targets for novel therapeutic schemes through alteration of methylation levels and miRNA expression.

Based on the proposed method, we identified not only the core miRNAs but also the core proteins and core target genes in signaling pathways by comparing different core GENs of the upper or lower progression paths in Figure 1(a). Normal liver cells provide a standard system for the toleration of intrinsic perturbations such as methylation accumulation and miRNA dysregulation to maintain liver homeostasis, but genetic mutations and epigenetic dysregulations are still accumulated and exceed the tolerable allowance of normal liver cells, causing liver diseases and eventually HCC. Therefore, the network biomarkers found to interact with other partners in the core signaling pathways could contribute to the genetic and epigenetic dysfunctions of the corresponding biological functions through genetic mutation, DNA methylation accumulation, and miRNA dysregulation.

**Figure 8:**
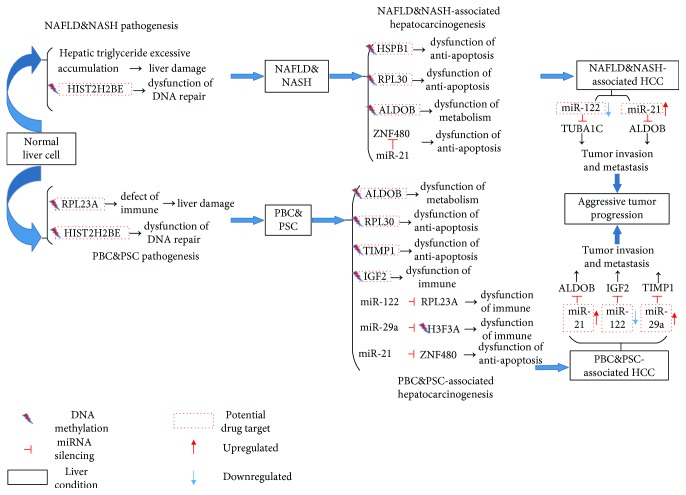
Schematic structure of the pathogenic and hepatocarcinogenic mechanisms of NAFLD&NASH and PBC&PSC.

According to our results, we shall further discuss the impact of miRNA regulation and DNA methylation on epigenetic mechanisms of pathogenesis and hepatocarcinogenesis from normal liver cells to HCC through NAFLD&NASH or PBC&PSC, as shown in Figures 6 and 7, respectively, as summarized in Figure 8.

### 3.1. Dysregulation of DNA Methylation and miRNA Regulation Contributes to Pathogenesis and Hepatocarcinogenesis from Normal Liver Cells to HCC through NAFLD&NASH or PBC&PSC

#### 3.1.1. Dysregulation of DNA Methylation and miR-21 Contributes to NAFLD&NASH Pathogenesis and NAFLD&NASH-Associated Hepatocarcinogenesis

Recently, several studies have indicated that NAFLD&NASH pathogenesis is associated with obesity and insulin resistance, facilitating liver damage through steatosis caused by excessive accumulation of hepatic triglycerides and more severe liver damage through subsequently induced oxidative stress [24]. Our results support the theory that oxidative stress can activate the MAPK signaling pathway to facilitate a series of functions such as DNA repair, apoptosis, and metabolism. In addition, we also determined that aberrant methylation can affect HIST2H2BE, facilitating the dysfunction of metabolism to progress from normal liver cells to NAFLD&NASH, as shown in Figure 6. Dysfunction of metabolism contributes to the accumulation of genetic mutations, which might cause more severe liver damage. It has been suggested that HIST2H2BE plays a central role in the progression of normal liver cell to NAFLD&NASH, so we suggested HIST2H2BE as a potential drug target that might overcome liver damage caused by excessive hepatic triglyceride accumulation.

After a brief discussion on NAFLD&NASH pathogenesis, we further discussed NAFLD&NASH hepatocarcinogenesis by abnormal miRNA regulation and aberrant methylation. In this study, we established that the change in the miRNA expression profile of miR-21 (*p* value ≤ 1.00 × 10^−3^) results in a significant change in the gene expression profile of *ZNF480* (*p* value ≤ 1.00 × 10^−3^) to progress from NAFLD&NASH to HCC, as shown in Figure 6. Recently, it has been reported that miR-21 is the first central oncomir to be associated with malignant cell proliferation, invasion, and metastasis in the carcinogenesis of a variety of cancers [29]. For instance, miR-21 can silence phosphatase and tensin homolog (PTEN), which is a tumor suppressor observed in HCC [29]. It not only reflects liver damage but also regulates other biological processes such as triglyceride and cholesterol metabolism by silencing target genes [30, 31] and the upregulation of ZNF480 resulting in tumor cell death [32]. Furthermore, our finding also demonstrated that epigenetic regulations, including DNA methylation of *HSPB1*, *ALDOB*, and *RPL30*, led to changes in the DNA methylation profiles of *HSPB1* (*p* value ≤ 1.00 × 10^−3^), *ALDOB* (*p* value ≤ 0.85), and *RPL30* (*p* value ≤ 1.00 × 10^−3^), which resulted in significant changes in the gene expression profiles of *HSPB1* (*p* value ≤ 1.00 × 10^−3^), *ALDOB* (*p* value ≤ 1.5 × 10^−2^), and *RPL30* (*p* value ≤ 1.00 × 10^−3^) in the progression from NAFLD&NASH to HCC, as shown in Figure 6. In addition, it has been proposed that the dysregulations of HSPB1, ALDOB, and RPL30 are sufficient to trigger tumor progression [33, 34]. Therefore, we suggested that the epigenetic dysregulation of miR-21 and DNA methylation causes pathogenesis and hepatocarcinogenesis from the upper progression path to HCC, as shown in Figure 8 with more detailed discussion in the sequel.

#### 3.1.2. Dysregulation of DNA Methylation, miR-21, miR-122, and miR-29a Contributes to PBC&PSC Pathogenesis and PBC&PSC-Associated Hepatocarcinogenesis

Based on our results from PBC&PSC pathogenesis, we identified that the DNA methylation profile changes for *RPL23A* and *HIST2H2BE* result in dysfunctions in the autoimmune and DNA repair response to progression from normal liver cells to PBC&PSC and give rise to the dysregulation of RYR2 through the WNT and the MAPK signaling pathways, to facilitate defects in autoimmunity as shown in Figure 7. Intriguingly, our results showed that PBC&PSC are associated with autoimmune dysfunction [35, 36]. It has been suggested that RPL23A and HIST2H2BE play a central role in the progression from normal liver cells to PBC&PSC, so we propose that RPL23A and HIST2H2BE are potential drug targets that might overcome the defects in autoimmunity and the subsequent dysfunction in metabolism.

After a brief discussion on PBC&PSC pathogenesis, we further discussed PBC&PSC hepatocarcinogenesis by abnormal miRNA regulation and aberrant methylation. We identified that the expression changes in miR-21 (*p* value ≤ 1.00 × 10^−3^), miR-122 (*p* value ≤ 1.4 × 10^−2^), and miR-29a (*p* value ≤ 1.00 × 10^−3^) contribute to the significant gene expression changes in *ALDOB* (*p* value ≤ 0.72 × 10^−1^), *RPL23A* (*p* value ≤ 1.00 × 10^−3^), and *H3F3A* (*p* value ≤ 1.00 × 10^−3^) between PBC&PSC and HCC, as shown in Figure 7. Recently, it has been reported that miR-122 is the most common miRNA; it is estimated to make up 70% of the total miRNA complement. It serves as an essential regulator and is involved in development, differentiation, and homeostasis, as well as the metabolism of glucose and lipids in the liver [1, 24]. Moreover, the loss of miR-122 has been associated with migration, invasion, and tumorigenesis, which could cause liver diseases and eventually HCC [1]. Consequently, the restoration of miR-122 represents antitumorigenesis functions that slow down the progression of both liver disease and HCC, which has been investigated and demonstrated in animal models [37]. Furthermore, a recent study has indicated that miR-29a might repress tumors formed by promoted apoptosis [38] and miR-29a is considered a potential target of therapy in autoimmune connective tissue disease [39]. Our findings demonstrated that epigenetic regulations, including DNA methylation of *RPL30*, *ALDOB*, *IGF2*, and *TIMP1*, led to changes in the DNA methylation profiles of *RPL30* (*p* value ≤ 1.00 × 10^−3^), *ALDOB* (*p* value ≤ 0.83), *IGF2* (*p* value ≤ 1.00 × 10^−3^), and *TIMP1* (*p* value ≤ 1.00 × 10^−3^), which contributed to the significant changes in the gene expression profiles of *RPL30* (*p* value ≤ 1.00 × 10^−3^), *ALDOB* (*p* value ≤ 7.2 × 10^−2^), *IGF2* (*p* value ≤ 1.1 × 10^−2^), and *TIMP1* (*p* value ≤ 1.00 × 10^−3^) to progress from PBC&PSC to HCC, as shown in Figure 7. In addition, it has been proposed that the dysregulation of DNA methylation of *RPL30*, *ALDOB*, *IGF2*, and *TIMP1* is sufficient to trigger tumor progression [33, 34, 40]. Abnormal miR-29a and hypomethylation contribute to the dysregulation of H3F3A, which could lead to tumorigenesis [41]. In summary, we suggested that the epigenetic dysregulation of miR-21, miR-122, miR-29a, and DNA methylation could cause pathogenesis and hepatocarcinogenesis in the lower progression path to HCC, as shown in Figure 8 with more detailed discussion in the sequel.

#### 3.1.3. Dysregulation of miR-21, miR-122, and miR-29a Contributes to Further Liver Damage in Patients with NAFLD&NASH or PBC&PSC

In this study, we confirmed that abnormal miR-21 silences ALDOB and abnormal miR-122 silences TUBA1C in the mechanisms of HCC progression, as shown in Figure 6. It has been proposed that TUBA1C is associated with tumor cell death and cell proliferation [42], and aberrant expression of ALDOB contributes to tumor metastasis, which is involved in the initiation and development of HCC [33, 43]. We also confirmed that abnormal miR-29a silences IGF2 and abnormal miR-122 silences TIMP1 in the mechanism of HCC progression, as shown in Figure 7. The dysregulation of IGF2 and TIMP1 was significantly realized in HCC. It has been reported that the dysregulation of IGF2 and TIMP1 results in tumor invasion and metastasis [40]. Therefore, we suggested that the dysregulation of miR-21, miR-29a, and miR-122 contributes to the dysregulation of TUBA1C, ALDOB, IGF2, and TIMP1, which might lead to tumor invasion and metastasis in the development of HCC, facilitating further aggressive tumor progression, as shown in Figure 8 with more detailed discussion in the sequel.

In brief, an increasing number of studies focus on epigenetic regulations that provide novel therapies from the perspective of epigenetic alterations. It has been proposed that the impact of miRNA regulation and methylation on different liver diseases and the mechanisms of progression from liver diseases to HCC are caused by epigenetic multiregulation. For instance, our results demonstrated that miR-122 and miR-21 might serve as epigenetic biomarkers in the upper progression path in Figure 8 and further provide potential drug targets for novel therapies through these two miRNAs and methylation of the target genes. Although the progression mechanism of the lower progression path is similar to that of the upper progression path in Figure 8, detailed progression mechanisms are differential. Hence, we identified miR-122, miR-21, and miR-29a as potential prognostic biomarkers for potential drug targets in the lower progression path in Figure 8. Interestingly, miR-122 and miR-21 may provide novel potential therapeutic targets for slowing down further liver damage in patients with NAFLD&NASH in Figure 8 and for predicting and treating HCC. PBC&PSC pathogenesis relates to autoimmune dysfunction and is different from NAFLD&NASH pathogenesis, which relates to metabolism dysfunction. Therefore, we suggested that miR-29a provides a novel potential therapeutic target for the restoration of aberrant immune response to treat PBC&PSC-developed HCC, as well as miR-122 and miR-21 in Figure 8. The results are supported by recent studies associated with miRNA regulation in the immune system [44].

Finally, the identified network biomarkers for preventing the hepatocarcinogenesis of NAFLD&NASH and PBC&PSC are shown in Table 1 (all potential drugs ranked in Table S3A).

## 4. Materials and Methods

The proposed methodology to identify the core signaling pathways and the network biomarkers of NAFLD&NASH pathogenesis, NAFLD&NASH-associated hepatocarcinogenesis, PBC&PSC pathogenesis, and PBC&PSC-associated hepatocarcinogenesis for preventing the progression of NAFLD&NASH or PBC&PSC is summarized in the flowchart in Figure 1(b).

### 4.1. Big Data Mining and Preprocessing of mRNA Expression Data and Its Corresponding DNA Methylation Profiles for Liver Diseases and HCC

In order to identify the real GENs of liver cells from the four kinds of patients including normal, NAFLD&NASH, PBC&PSC, and HCC patients, the simultaneously measured genome-wide mRNA expression data and DNA methylation profiles in each sample of the patients with one of the liver conditions were needed. Also, the candidate GENs obtained from biological or computational experiments in human cells were required. In this study, we used the microarray data with its corresponding DNA methylation profiles of 134 samples in patients with normal (62 samples), NAFLD&NASH (47 samples), and PBC&PSC (25 samples) conditions from the National Center for Biotechnology Information (NCBI) Gene Expression Omnibus (GEO) database (GSE61260) provided by Horvath et al. [45] and used the NGS data and its corresponding DNA methylation profiles of 369 samples in patients with HCC, obtained from the Cancer Genome Atlas (TCGA) (https://genome-cancer.ucsc.edu/). NGS data, microarray data, and DNA methylation profiles were measured using the Illumina HiSeq platform, the HuGen1.1ST platform, and the Illum450K platform, respectively.

Additionally, we considered that the candidate GEN in human cells includes the candidate PPIs and gene and miRNA regulations. According to the candidate PPIs from the Biological General Repository for Interaction Datasets (BioGRID) database [46], the gene regulations from the Integrated Transcription Factor Platform (ITFP) database, the Human Transcriptional Regulation Interactions (HTRI) database, and the TRANScription FACtor database (TRANSFAC) [47], and the miRNA regulations from TargetScanHuman database [48], we obtained 168,970 PPI pairs, 152,828 gene regulations, and 247,743 miRNA repressions in the candidate GEN of human cells. Because the candidate GEN of human cells contains all possible associations obtained from experimental and computational results, which contain many false positives, we need to construct the stochastic interactive/regulatory models of the candidate GEN in human cells to characterize the cellular mechanisms in the network. The real GEN of each liver condition can then be obtained by pruning away false positives in the candidate GEN through a system identification method and a system order detection scheme in the stochastic models using the genome-wide mRNA expression data and its corresponding DNA methylation profiles.

To integrate the big data, including genome-wide mRNA expression data, its corresponding DNA methylation profiles, and the candidate interactions/regulations, from several databases, we used Matlab's text-file and string manipulation tools in text mining to unify the gene name based on the gene symbols in the NCBI Entrez Gene database.

### 4.2. Constructing the Stochastic Models of the GEN in Human Cells

We constructed the stochastic models of the candidate GEN in human cells to characterize the molecular mechanisms, including PPIs, transcriptional regulations, miRNA repressions, DNA methylation, and stochastic noises due to the modeling residue and the fluctuation of genes. The molecular mechanisms of the *i*th protein in the PPIN of the GEN in the cells of the *n*th patient sample can be described by the stochastic protein interactive model as follows:

(1) $$\begin{array}{c} y_{i}\left[n\right]=\overset{N_{i}}{\underset{j=1,j\neq i}{\sum }}a_{ij}y_{i}\left[n\right]y_{j}\left[n\right]+h_{i}+v_{i}\left[n\right],\;\text{for }i=1,\ldots ,N,\;n=1,\ldots ,L,\;h_{i}\geq 0, \end{array}$$

where *y*_*i*_[*n*], *y*_*j*_[*n*], and *v*_*i*_[*n*] represent the mRNA expression levels of the *i*th protein and its *j*th interactive protein and the stochastic noise of the *i*th protein due to model uncertainty or the fluctuation of expressions in patient *n*, respectively; *L*, *N*, and *N*_*i*_ indicate the number of patients, proteins, and proteins interacting with the *i*th protein in the candidate PPIN, respectively; and *a*_*ij*_ and *h*_*i*_ denote the interaction ability between the *i*th protein and its *j*th interactive protein and the basal level of the *i*th mRNA expression, respectively. The term *y*_*i*_[*n*]*y*_*j*_[*n*], the product of the concentrations of two interactive proteins, can represent the probability of molecular interaction between two proteins of the complex protein interaction. The probability of molecular interaction between two proteins has been also modeled as the probability of molecule collision by a nonlinear multiplication scheme [49, 50]. The molecular mechanisms in (1) of the *i*th protein in the PPIN of human cells in the *n*th patient sample include PPIs (∑_*j*=1,*j*≠*i*_^*N*_*i*_^ *a*_*ij*_ *y*_*i*_[*n*]*y*_*j*_[*n*]), the stochastic noise (*v*_*i*_[*n*]), and the basal levels of proteins (*h*_*i*_).

The molecular mechanisms of the *i*th gene in the GRN of the GEN in the cells of the *n*th patient sample can be described by the stochastic gene regulatory model as follows:

(2) $$\begin{array}{c} x_{i}\left[n\right]=\overset{K_{i}}{\underset{j=1,j\neq i}{\sum }}b_{ij}y_{j}\left[n\right]M_{i}\left[n\right]-\overset{V_{i}}{\underset{v=1}{\sum }}c_{iv}x_{i}\left[n\right]x_{v}^{miR}\left[n\right]M_{i}\left[n\right]+M_{i}\left[n\right]\kappa_{i}+\omega_{i}\left[n\right],\;\text{for }i=1,\ldots ,N,\;n=1,\ldots ,L,\;\kappa_{i},c_{iv}\geq 0, \end{array}$$

where *x*_*i*_[*n*], *y*_*j*_[*n*], *x*_*v*_^*miR*^[*n*], and *ω*_*i*_[*n*] indicate the expression levels of the *i*th target gene, its *j*th regulatory TF, its *v*th regulatory miRNA, and its stochastic noise, which results from model uncertainty or the fluctuation of expressions in GRN, in patient *n*, respectively; *K*_*i*_ and *V*_*i*_ represent the number of TFs and miRNAs binding to the *i*th gene based on candidate GEN in human cells, respectively; *κ*_*i*_ denotes the basal level of the *i*th gene; *b*_*ij*_ and *c*_*i ***v**_ indicate the transcriptional regulatory ability from the *j*th TF to the *i*th gene and the posttranscriptional regulatory ability of the *v*th miRNA to inhibit the *i*th gene (−*c*_*iv*_ ≤ 0), respectively; and *M*_*i*_[*n*] represents methylation regulation of the *i*th gene, affecting the binding affinities of miRNAs, RNA polymerase, and TFs to the *i*th gene [51]. The effect of methylation on binding affinities of miRNAs, RNA polymerase, and TFs to the *i*th gene is expressed by the terms *c*_*iv*_ *x*_*i*_[*n*]*x*_*v*_^miR^[*n*]*M*_*i*_[*n*], *M*_*i*_[*n*]*κ*_*i*_, and *b*_*ij*_ *y*_*j*_[*n*]*M*_*i*_[*n*], respectively. The methylation regulation of the *i*th target gene *M*_*i*_[*n*] is defined as follows:

(3) $$\begin{array}{c} M_{i}\left[n\right]≕\frac{1}{1+{\left(m_{i}\left[n\right]/0.5\right)}^{2}}, \end{array}$$

where *m*_*i*_[*n*] denotes the DNA methylation profile of the *i*th gene in the *n*th patient sample. Because the numerical range of DNA methylation profiles *m*_*i*_[*n*] is between 0 and 1, the effect of DNA methylation on gene and miRNA regulations and basal levels is from 1 to 0.2. It means that if the DNA methylation profile of the *i*th gene increases, the binding affinities of miRNAs, RNA polymerase, and TFs to the *i*th gene decrease. The definition of methylation regulation in (3) avoids shutting down the bindings of miRNAs, RNA polymerase, and TFs to the *i*th gene by *m*_*i*_[*n*] = 1. The basal level (*κ*_*i*_) change between two liver conditions is also used to infer the effect of DNA methylation on the transcriptional regulation of the *i*th gene. The molecular mechanisms in (2) of the *i*th gene in the GRN of human cells in the *n*th patient sample include transcriptional regulations (∑_*j*=1,*j*≠*i*_^*K*_*i*_^ *b*_*ij*_ *y*_*j*_[*n*]*M*_*i*_[*n*]), miRNA repressions (−∑_*v*=1_^*V*_*i*_^ *c*_*iv*_ *x*_*i*_[*n*] · *x*_*v*_^miR^[*n*]*M*_*i*_[*n*]), the stochastic noise (*ω*_*i*_[*n*]), and the effects of RNA polymerase and DNA methylation (*M*_*i*_[*n*]*κ*_*i*_) on genes.

### 4.3. Identification of the Real GEN by Applying System Identification Method and System Order Detection Scheme to Prune False Positives in Candidate GEN Using Genome-Wide Expression Data and Its Corresponding DNA Methylation Profiles

After constructing the stochastic protein interactive model in (1) of PPIN and the stochastic gene regulatory model in (2) of GRN based on molecular mechanisms of human cells, we then applied the system identification method and system order detection scheme to the models using mRNA expression data and its corresponding DNA methylation profiles in normal liver, NAFLD&NASH, PBC&PSC, and HCC to identify their corresponding real GENs. In order to identify the parameters in (1) and (2) using mRNA expression data and its corresponding DNA methylation profiles, we rewrite (1) and (2) as the following regression forms (4) and (5), respectively:

(4) $$\begin{array}{c} y_{i}\left[n\right]=\left[y_{i}\left[n\right]y_{1}\left[n\right]\;\cdots \;y_{i}\left[n\right]y_{i-1}\left[n\right]\;y_{i}\left[n\right]y_{i+1}\left[n\right]\;\cdots \;y_{i}\left[n\right]y_{N_{i}}\left[n\right]\;1\right]\times \left[\begin{array}{c} a_{i1}\\ \vdots \\ a_{ii-1}\\ a_{ii+1}\\ \vdots \\ a_{{\text{iN}}_{i}}\\ h_{i} \end{array}\right]+v_{i}\left[n\right]=\phi_{i,PPIN}\left[n\right]\theta_{i,PPIN}+v_{i}\left[n\right],\;\text{for }i=1,\ldots ,N,\;n=1,\ldots ,L,\;h_{i}\geq 0, \end{array}$$

(5) $$\begin{array}{c} x_{i}\left[n\right]=\left[y_{1}\left[n\right]M_{i}\left[n\right]\;\cdots \;y_{i-1}\left[n\right]M_{i}\left[n\right]\;y_{i+1}\left[n\right]M_{i}\left[n\right]\cdots y_{K_{i}}\left[n\right]M_{i}\left[n\right]\;x_{i}\left[n\right]x_{1}^{miR}\left[n\right]M_{i}\left[n\right]\;\cdots \;x_{i}\left[n\right]x_{V_{i}}^{miR}\left[n\right]M_{i}\left[n\right]\;M_{i}\left[n\right]\right]\times \left[\begin{array}{c} b_{i1}\\ \vdots \\ b_{ii-1}\\ b_{ii+1}\\ \vdots \\ b_{{iK}_{i}}\\ -c_{i1}\\ \vdots \\ -c_{{iV}_{i}}\\ \kappa_{i} \end{array}\right]+\omega_{i}\left[n\right]=\phi_{i,GRN}\left[n\right]\theta_{i,GRN}+\omega_{i}\left[n\right],\;\text{for }i=1,\ldots ,N,\;n=1,\ldots ,L,\;\kappa_{i},c_{iv}\geq 0, \end{array}$$

where *ϕ*_*i*,PPIN_[*n*] and *ϕ*_*i*,GRN_[*n*] denote the regression vectors, which consist of mRNA expression data and its corresponding DNA methylation profiles, and *θ*_*i*,PPIN_ and *θ*_*i*,GRN_ indicate the parameter vectors, which consist of protein interaction abilities, transcriptional and posttranscriptional regulatory abilities and basal levels, i.e., linking weights of GEN.

Moreover, we rewrite the regression forms in (4) and (5) as the following matrix forms for all *L* patients, respectively:

(6) $$\begin{array}{c} Y_{i}=\Phi_{i,PPIN}\theta_{i,PPIN}+V_{i},\;\text{for }i=1,\ldots ,N,\;h_{i}\geq 0,\\ X_{i}=\Phi_{i,GRN}\theta_{i,GRN}+W_{i},\;\text{for }i=1,\ldots ,N,\;\kappa_{i},c_{iv}\geq 0, \end{array}$$

where

(7) $$\begin{array}{c} Y_{i}=\left[\begin{array}{c} y_{i}\left[1\right]\\ \vdots \\ y_{i}\left[L\right] \end{array}\right],\\ X_{i}=\left[\begin{array}{c} x_{i}\left[1\right]\\ \vdots \\ x_{i}\left[L\right] \end{array}\right],\\ \Phi_{i,PPIN}=\left[\begin{array}{c} \phi_{i,PPIN}\left[1\right]\\ \vdots \\ \phi_{i,PPIN}\left[L\right] \end{array}\right],\\ \Phi_{i,GRN}=\left[\begin{array}{c} \phi_{i,GRN}\left[1\right]\\ \vdots \\ \phi_{i,GRN}\left[L\right] \end{array}\right],\\ V_{i}=\left[\begin{array}{c} v_{i}\left[1\right]\\ \vdots \\ v_{i}\left[L\right] \end{array}\right],\\ W_{i}=\left[\begin{array}{c} \omega_{i}\left[1\right]\\ \vdots \\ \omega_{i}\left[L\right] \end{array}\right]. \end{array}$$

Therefore, the parameter vectors *θ*_*i*,PPIN_ and *θ*_*i*,GRN_ can then be obtained by solving the following constrained least square parameter estimation problems, respectively [52]:

(8) $$\begin{array}{c} \underset{\theta_{i,PPIN}}{\min}\;\frac{1}{2}{\left\|\Phi_{i,PPIN}\theta_{i,PPIN}-Y_{i}\right\|}_{2}^{2} \end{array}$$

(9) $$\begin{array}{c} \underset{\theta_{i,GRN}}{\min}\;\frac{1}{2}{\left\|\Phi_{i,GRN}\theta_{i,GRN}-X_{i}\right\|}_{2}^{2} \end{array}$$

where the accuracies of the parameter estimations in (8) and (9) were given by the following error estimations ${\hat{\sigma }}_{i}^{2}={\left(Y_{i}-\Phi_{i,PPIN}{\hat{\theta }}_{i,PPIN}\right)}^{T}\left(Y_{i}-\Phi_{i,PPIN}{\hat{\theta }}_{i,PPIN}\right)/L$and ${\hat{\sigma }}_{i}^{2}={\left(X_{i}-\Phi_{i,GRN}{\hat{\theta }}_{i,GRN}\right)}^{T}\left(X_{i}-\Phi_{i,GRN}{\hat{\theta }}_{i,GRN}\right)/L$, respectively, and ${\hat{\theta }}_{i,PPIN}$ and ${\hat{\theta }}_{i,GRN}$ represent the identified parameters. The solutions of the above problems, in which inequality constraints can guarantee the negative effect of miRNA regulation on genes and the positive basal levels of genes/proteins, can be obtained by applying the system identification method in MATLAB optimization toolbox using genome-wide mRNA expression data and DNA methylation profiles based on a reflective Newton method for minimizing a quadratic function [53].

Since genome-wide expression measurement of protein behaviors in normal liver, NAFLD&NASH, PBC&PSC, and HCC has not been realized yet and gene expressions are proportional to their corresponding proteins, in which 73% variance of protein abundance can be explained by mRNA abundance [54], the mRNA expressions can replace protein expressions for the above constrained least square parameter estimation problems in (8) and (9). Because mRNA expression data (GSE61260) and its corresponding DNA methylation profiles (GSE61258) from normal liver, NAFLD&NASH, and PBC&PSC have been reported and NGS data and its corresponding DNA methylation profiles from HCC have been also proposed in TCGA, i.e., *Y*_*i*_, *X*_*i*_, Φ_*i*,PPIN_, and Φ_*i*,GRN_ in (8) and (9) are all available; protein interaction abilities, transcriptional and posttranscriptional regulatory abilities, and basal levels, i.e., linking weights of GEN, in *θ*_*i*,PPIN_ and *θ*_*i*,GRN_ can then be identified.

Because the candidate GEN obtained from biological or computational experiments in human cells contains all putative interactions in PPIN in (8) and regulations in GRN in (9), in order to prune the false-positive protein interaction abilities and transcriptional and posttranscriptional regulatory abilities, i.e., links of GEN, based on the genome-wide expression data in normal liver, NAFLD&NASH, PBC&PSC, and HCC, we applied a system order detection scheme, AIC, to the system identification method in solving (8) and (9) to detect the real system order (or real links) by minimizing the following AIC value [55, 56]:

(10) $$\begin{array}{c} AIC\left(\Delta_{i}\right)=\log\left({\hat{\sigma }}_{i}^{2}\right)+\frac{2\left(\Delta_{i}\right)}{L}, \end{array}$$

where Δ_*i*_ denotes the number of parameters, i.e., Δ_*i*_ = *N*_*i*_ + 1 in the estimation problem of the PPIN model (8) and Δ_*i*_ = *K*_*i*_ + *V*_*i*_ + 1 in the estimation problem of the GRN model (9), and ${\hat{\sigma }}_{i}^{2}$ is the estimated residual error obtained from the system identification method, i.e., ${\hat{\sigma }}_{i}^{2}={\left(Y_{i}-\Phi_{i,PPIN}{\hat{\theta }}_{i,PPIN}\right)}^{T}\left(Y_{i}-\Phi_{i,PPIN}{\hat{\theta }}_{i,PPIN}\right)/L$ and ${\hat{\sigma }}_{i}^{2}={\left(X_{i}-\Phi_{i,GRN}{\hat{\theta }}_{i,GRN}\right)}^{T}\left(X_{i}-\Phi_{i,GRN}{\hat{\theta }}_{i,GRN}\right)/L$ in the estimation problems of the PPIN model (8) and the GRN model (9), respectively. In this study, we applied stepwise selection, a combination of the forward selection and backward elimination techniques until none improves the minimization problem in (10), to the candidate GEN. According to the theory of system identification [55, 57], the true system order Δ_*i*_^∗^ (or the true number of links) of the real GEN could minimize AIC(*∆*_*i*_) in (10). After getting the true system order Δ_*i*_^∗^ by minimizing AIC(*∆*_*i*_) in (10), we could prune the false-positive interactions and regulations of the candidate GEN by deleting the insignificant links out of true system order Δ_*i*_^∗^ detected by AIC. Moreover, we applied Student's *t*-test to the real GEN to calculate the *p* value of each interaction or regulatory ability (or each link of the real GEN) under the null hypothesis *H*_0_ : *a*_*im*_ = 0 or *H*_0_ : *b*_in_ = 0 [58]. In this study, we applied random permutation to the sample data to generate 1000 random sets of the data. For example, if none of the 1000 permutation values exceeds the test statistic, *p* value is less than or equal to 10^−3^. The *p* value of each interaction or regulatory ability is used to support our findings.

According to the data in GSE61260 and GSE61258, we obtained genome-wide mRNA expression data and its corresponding DNA methylation profiles in normal liver from 62 patients, NAFLD&NASH from 47 patients, and PBC&PSC from 25 patients. Additionally, according to the data in TCGA, we obtained genome-wide mRNA expression data and its corresponding DNA methylation profiles from HCC in 369 patients. Therefore, we can identify four GENs of liver cells for normal liver, NAFLD&NASH, PBC&PSC, and HCC, respectively.

In brief, we first identified the interaction and regulatory abilities, including *a*_*ij*_, *b*_*ij*_, and *c*_*iv*_ in (1) and (2), of the candidate GEN in each of four liver conditions by applying the system identification method using the genome-wide expression data. We then applied AIC to prune the false-positive interactions and regulations in the candidate GEN and finally obtain the real GEN according to the gene expression data and its DNA methylation profiles in each of liver conditions. We applied Student's *t*-test to the real GEN to calculate the *p* value of each interaction or regulatory ability in each of four liver conditions to support our findings.

### 4.4. Extracting the Core GEN from the Genome-Wide Real GEN via PNP

After identifying the genome-wide real GENs of four liver conditions using gene expression data and DNA methylation profiles to prune the false-positive interactions and regulations in the candidate GEN by applying a system order detection scheme, these genome-wide GENs are still very complex and not easy to get an insight into their pathogenic and hepatocarcinogenic mechanisms in four liver conditions. We then apply the PNP method to determine the core proteins/genes/miRNAs, which compose the core GENs from the genome-wide real GENs of four liver conditions. The PPIs, gene regulations, miRNA inhibitions, and DNA methylation of the real GENs are described by the following models:

(11) $$\begin{array}{c} y_{i}\left[n\right]=\underset{j\in N_{P}}{\sum }{\hat{a}}_{ij}y_{i}\left[n\right]y_{j}\left[n\right]+{\hat{h}}_{i}+v_{i}\left[n\right],\;\text{for }i=1,\ldots ,N,\;n=1,\ldots ,L, \end{array}$$

(12) $$\begin{array}{c} x_{i}\left[n\right]=\underset{j\in K_{G}}{\sum }{\hat{b}}_{ij}y_{j}\left[n\right]M_{i}\left[n\right]-\underset{v\in V_{G}}{\sum }{\hat{c}}_{iv}x_{i}\left[n\right]x_{v}^{miR}\left[n\right]M_{i}\left[n\right]+M_{i}\left[n\right]{\hat{\kappa }}_{i}+\omega_{i}\left[n\right],\;\text{for }i=1,\ldots ,N,\;n=1,\ldots ,L, \end{array}$$

where *N*_*P*_, *K*_*G*_, and *V*_*G*_ indicate the real number of PPIs, gene regulations, and miRNA inhibitions, respectively, and ${\hat{a}}_{ij}$, ${\hat{b}}_{ij}$, and ${\hat{c}}_{iv}$ represent the estimated interaction abilities of proteins, the estimated regulatory abilities of TFs, and the estimated posttranscriptional regulatory abilities of miRNAs by applying a system identification method and a system order detection scheme to (8) and (9), respectively.

We generally integrated ${\hat{a}}_{ij}$, ${\hat{b}}_{ij}$, and ${\hat{c}}_{iv}$, linking weights of the real GEN, from (11) and (12) as the following network structure matrix *H*:

(13) $$\begin{array}{c} H={\left[\begin{array}{ccccccccccccccc} {\hat{a}}_{11} & \cdots & {\hat{a}}_{1j} & \cdots & {\hat{a}}_{1N} & {\hat{b}}_{11} & \cdots & {\hat{b}}_{1j} & \cdots & {\hat{b}}_{1N} & -{\hat{c}}_{11} & \cdots & -{\hat{c}}_{1v} & \cdots & -{\hat{c}}_{1V}\\ \vdots & \ddots & \vdots & \ddots & \vdots & \vdots & \ddots & \vdots & \ddots & \vdots & \vdots & \ddots & \vdots & \ddots & \vdots \\ {\hat{a}}_{i1} & \cdots & {\hat{a}}_{ij} & \cdots & {\hat{a}}_{\text{iN}} & {\overset{\wedge }{b}}_{i1} & \cdots & {\hat{b}}_{ij} & \cdots & {\hat{b}}_{\text{iN}} & -{\hat{c}}_{i1} & \cdots & -{\hat{c}}_{iv} & \cdots & -{\hat{c}}_{iV}\\ \vdots & \ddots & \vdots & \ddots & \vdots & \vdots & \ddots & \vdots & \ddots & \vdots & \vdots & \ddots & \vdots & \ddots & \vdots \\ {\hat{a}}_{N1} & \cdots & {\hat{a}}_{Nj} & \cdots & {\hat{a}}_{NN} & {\hat{b}}_{N1} & \cdots & {\hat{b}}_{Nj} & \cdots & {\hat{b}}_{NN} & -{\hat{c}}_{N1} & \cdots & -{\hat{c}}_{Nv} & \cdots & -{\hat{c}}_{NV} \end{array}\right]}^{T}. \end{array}$$

If a link between any two members of genes, proteins, and miRNAs in the real GEN is disconnected, its corresponding element in *H* is set to zero. PNP is a network structure projection method based on singular value decomposition (SVD) as follows [56]:

(14) $$\begin{array}{c} H={QSR}^{T}, \end{array}$$

where *Q* ∈ ℛ^(2*N* + *V*)×*N*^; *H* = [*h*_1_ ⋯ *h*_*k*_ ⋯ *h*_2N+*V*_]^T^ ∈ ℛ^(2*N* + *V*)×*N*^; *h*_*k*_ ∈ ℛ^1×*N*^; *R* = [*r*_1_ ⋯ *r*_*m*_ ⋯ *r*_*N*_] ∈ ℛ^*N*×*N*^; *r*_*m*_ ∈ ℛ^*N*^; *S* is a diagonal matrix such as *S* = diag(*s*_1_, ⋯, *s*_*m*_, ⋯, *s*_*N*_), which consists of *N* non-negative singular values of *H* with descending order *s*_1_ ≥ ⋯≥*s*_*m*_ ≥ ⋯≥*s*_*N*_; and diag(*s*_1_, *s*_2_) denotes the diagonal matrix of *s*_1_ and *s*_2_, i.e., $\left[\begin{array}{cc} s_{1} & 0\\ 0 & s_{2} \end{array}\right]$. We then defined the eigenexpression fraction (*E*_*m*_) as follows:

(15) $$\begin{array}{c} E_{m}=\frac{s_{m}^{2}}{\sum_{m=1}^{N}s_{m}^{2}}. \end{array}$$

The principal components to satisfy ∑_*m*=1_^*M*^ *E*_*m*_ ≥ 0.85 with the minimal *M* are selected as the top *M* singular vectors of *R* in (14) to determine the core proteins/genes/miRNAs. Furthermore, the genome-wide real GEN in *H* is projected to the top *M* singular vector of *R* as follows:

(16) $$\begin{array}{c} p\left(k,m\right)=h_{k}r_{m},\;\text{for }k=1,\ldots ,2N+V,\;m=1,\ldots ,M. \end{array}$$

We can then obtain the projection value from each member of the real GEN onto the top *M* singular vectors (i.e., principal network structure) by applying the 2-norm to (16) as the dependent score *D*(*k*) of each member as follows:

(17) $$\begin{array}{c} D\left(k\right)={\left(\overset{M}{\underset{m=1}{\sum }}{\left[p\left(k,m\right)\right]}^{2}\right)}^{1/2},\;\text{for }k=1,\ldots ,2N+V. \end{array}$$

According to the projection value, i.e., dependent score *D*(*k*), from each member of the real GEN onto the top *M* singular vectors of *H* based on their respective interactions or regulatory abilities, ${\hat{a}}_{ij}$, ${\hat{b}}_{ij}$, and ${\hat{c}}_{iv}$, we can finally calculate the dependent score *D*(*k*) of proteins (*k* = 1,…, *N*), genes (*k* = *N* + 1,…, 2*N*) and miRNAs (*k* = 2*N* + 1,…, 2*N* + *V*) to the principal network structure of the genome-wide real GEN. If *D*(*k*) is close to zero, it means that the *k*th member of the real GEN is independent of the top *M* singular vectors (or principal network structure). Otherwise, a member of the real GEN with higher *D*(*k*) is more important for the principal network structure of the real GEN. In order to determine the core proteins/genes/miRNAs, which compose the core GEN from a genome-wide real GEN, we choose the top 5% proteins in *D*(*k*) for *k* = 1,…, *N* and their connecting genes and miRNAs not only to constitute the core GEN but also to constitute the complete connections in signal transduction; i.e., the top 5% proteins just could construct a complete signaling cascade from receptors to TFs. Therefore, we applied PNP to the real GENs of normal liver, NAFLD&NASH, PBC&PSC, and HCC to obtain the core GENs of them. By comparing the core GENs between two liver conditions, we further extracted signaling pathways and network biomarkers from the core GENs to investigate the cellular mechanisms of pathogenesis and hepatocarcinogenesis for preventing the progression of liver damage.

## 5. Conclusion

In this study, we focused on constructing the corresponding signaling pathways involved in the core GENs to further investigate cellular mechanisms of progression from normal liver cells to HCC through NAFLD&NASH or PBC&PSC in Figure 1(a), based on miRNA regulation and epigenetic regulation in GENs. To begin with, we proposed a novel approach to constructing the genome-wide candidate GEN using big database mining. We then pruned the false-positive regulatory interactions using a system order detection scheme to identify the genome-wide real GEN. We also used PNP to construct core GENs for different liver diseases. Since core GENs are complicated, mechanism interpretation is difficult. Therefore, we extracted core networks for each progression stage in Figure 1(a) by comparing different core GENs and further submitted core networks to the KEGG database to analyze relevant signaling pathways and to investigate cellular mechanisms of progression from normal liver cells to HCC through NAFLD&NASH or PBC&PSC. Based on the core signaling pathways, we also realized genetic and epigenetic regulatory mechanisms of progression from normal liver cells to HCC through NAFLD&NASH or PBC&PSC using KEGG and GO database mining. Finally, we observed that some miRNA regulations and epigenetic regulations fulfilled essential roles in the mechanisms of progression from normal liver cells to HCC through the upper or the lower progression paths in Figure 8. Hence, our results show key network biomarkers of miRNA regulation and methylation in the mechanisms of progression. Therefore, network biomarkers could also be developed based on potential drug targets for the two progression paths of pathogenesis and hepatocarcinogenesis in Figure 8.

## Figures and Tables

**Table 1:** Network biomarkers for preventing the hepatocarcinogenesis of NAFLD&NASH and PBC&PSC.

	NAFLD&NASH to HCC	PBC&PSC to HCC
The highly activated network biomarkers for potential inhibition strategy of drug design	ACTA2, AIFM1, APP, EGFR, GRB2, NEDD8, SHC1, TUBA1C, TUBB6, IL4R, GPR37, FRAT2, HIST2H2BE, HSPB1, ZNF480, TIMP1, ALDOB, ZYX, YBX1, TP53, STAT5A, AR, TELO2, PXN, TBP, REPIN1, HSF1, ETS1, STUB1, RFC5, RPL30, TRMT1, GSK3B, UBC, ALB, and MIR21	ACTB, ADRB2, APP, CSNK2A1, EGFR, HUWE1, LMNB1, PCK1, PPP2CA, SMAD5, TSC22D1, ESR1, PGR, RYR2, FRAT2, HIST2H2BE, ZNF480, H3F3A, TIMP1, ALDOB, IGF2, AR, TP53, SRF, NRF1, STAT5A, RPL23A, SP1, SP3, CEBPA, BPTF, ETS1, MDC1, RFC5, RPL30, TRMT1, UBC, GSK3B, TF, and MIR21
The repressed network biomarkers for potential activation strategy of drug design	ALK and GATA1	CEBPE and GATA1

## Data Availability

The data supporting the results of this article are included within the article and its additional files.

## Abbreviations

AIC: Akaike information criterion
aldolase B: ALDOB
APP: Amyloid precursor protein
AR: Androgen receptor
ATZ: Alpha-1 antitrypsin Z
BioGRID: Biological General Repository for Interaction Datasets
EGFR: Epidermal growth factor receptor
ETS1: V-Ets avian erythroblastosis virus E26 oncogene homolog 1
FRAT2: Frequently rearranged in advanced T-cell lymphomas 2
GEN: Genome-wide real genetic and epigenetic network
GEO: Gene expression omnibus
GO: Gene ontology
GRN: Gene regulatory network
H2be: Histone cluster 2
H3F3A: H3 histone family 3A
HCC: Hepatocellular carcinoma
HSPB1: Heat shock 27 kDa protein 1
HTRI: Human transcriptional regulation interactions
IGF2: Insulin-like growth factor 2
ITFP: Integrated transcription factor platform
KEGG: Kyoto encyclopedia of genes and genomes
NAFLD: Nonalcoholic fatty liver disease
NASH: Nonalcoholic steatohepatitis
NAFLD&NASH: Nonalcoholic fatty liver disease and nonalcoholic steatohepatitis
NCBI: National Center for Biotechnology Information
miRNA: MicroRNA
PBC: Primary biliary cirrhosis
PBC&PSC: Primary biliary cirrhosis and primary sclerosing cholangitis
PSC: Primary sclerosing cholangitis
PNP: Principal network projection
PPI: Protein-protein interaction
PPIN: Protein-protein interaction network
PTEN: Phosphatase and tensin homolog
RFC5: Replication factor C5
RPL30: Ribosomal protein L30
RYR2: Ryanodine receptor 2
SHC1: SHC-transforming protein 1
SVD: Singular value decomposition
TCGA: The Cancer Genome Atlas
TF: Transcription factor
TIMP1: TIMP metallopeptidase inhibitor 1
TRANSFAC: TRANScription FACtor database
TRMT1: tRNA methyltransferase 1
TUBA1C: Tubulin alpha 1c
UBC: Ubiquitin C
ZNF480: Zinc finger protein 480.

## Supplementary Materials

Supplementary Materials Figure S1: the genetic and epigenetic network (GEN) for normal liver cell. Figure S2: the genetic and epigenetic network (GEN) for NAFLD&NASH. Figure S3: the genetic and epigenetic network (GEN) for PBC&PSC. Figure S4: the genetic and epigenetic network (GEN) for HCC. Figure S5: the core genetic and epigenetic network (GEN) for normal liver cell 41. Figure S6: the core genetic and epigenetic network (GEN) for NAFLD&NASH. Figure S7: the core genetic and epigenetic network (GEN) for PBC&PSC. Figure S8: the core genetic and epigenetic network (GEN) for HCC. Table S1: the projection values of the 37 network biomarkers NAFLD&NASH pathogenesis and NAFLD&NASH-associated hepatocarcinogenesis. Table S2: the projection values of the 40 network biomarkers in PBC&PSC pathogenesis and PBC&PSC-associated hepatocarcinogenesis. Table S3: a list of ranked drugs to prevent hepatocarcinogenesis of NAFLD&NASH (A) and PBC&PSC (B).
