# Supplementary material for: Investigating Pathogenic and Hepatocarcinogenic Mechanisms from Normal Liver to HCC by Constructing Genetic and Epigenetic Networks via Big Genetic and Epigenetic Data Mining and Genome-Wide NGS Data Identification
Source: Dis Markers. 2018 Sep 23;2018:8635329. doi: 10.1155/2018/8635329 (PMC6174771; doi:10.1155/2018/8635329)
Supplement: Supplementary Materials — Figure S1: the genetic and epigenetic network (GEN) for normal liver cell. Figure S2: the genetic and epigenetic network (GEN) for NAFLD&NASH. Figure S3: the genetic and epigenetic network (GEN) for PBC&PSC. Figure S4: the genetic and epigenetic network (GEN) for HCC. Figure S5: the core genetic and epigenetic network (GEN) for normal liver cell 41. Figure S6: the core genetic and epigenetic network (GEN) for NAFLD&NASH. Figure S7: the core genetic and epigenetic network (GEN) for PBC&PSC. Figure S8: the core genetic and epigenetic network (GEN) for HCC. Table S1: the projection values of the 37 network biomarkers NAFLD&NASH pathogenesis and NAFLD&NASH-associated hepatocarcinogenesis. Table S2: the projection values of the 40 network biomarkers in PBC&PSC pathogenesis and PBC&PSC-associated hepatocarcinogenesis. Table S3: a list of ranked drugs to prevent hepatocarcinogenesis of NAFLD&NASH (A) and PBC&PSC (B). [file 8635329.f1.docx]

**Supplementary Materials**


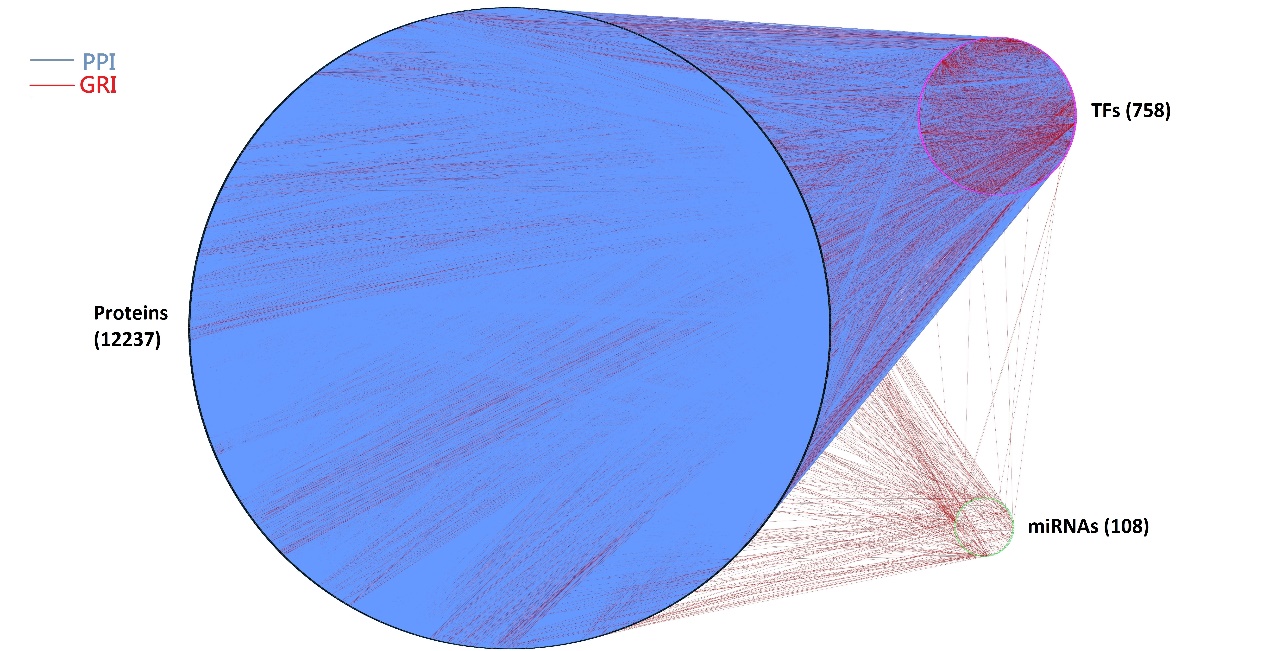


Figure S1. The genetic-and-epigenetic network (GEN) for normal liver cell

The left circle represents the proteins (12237), the upper right circle represents the TFs (758) and the under right circle represents the miRNAs (108) in the GEN for normal liver cell. Blue line represents protein-protein interaction (PPI) and red line represents gene regulatory information (GRI); PPIs are between protein circle and TF circle. GRIs are divided into the regulation of transcription factors on target genes and the regulation of miRNAs on target genes. The regulations of transcription factors on target genes are between TF circle and protein circle, and the regulations of miRNAs on target genes are between TF & protein circle and miRNA circle in the GEN for normal liver cell.


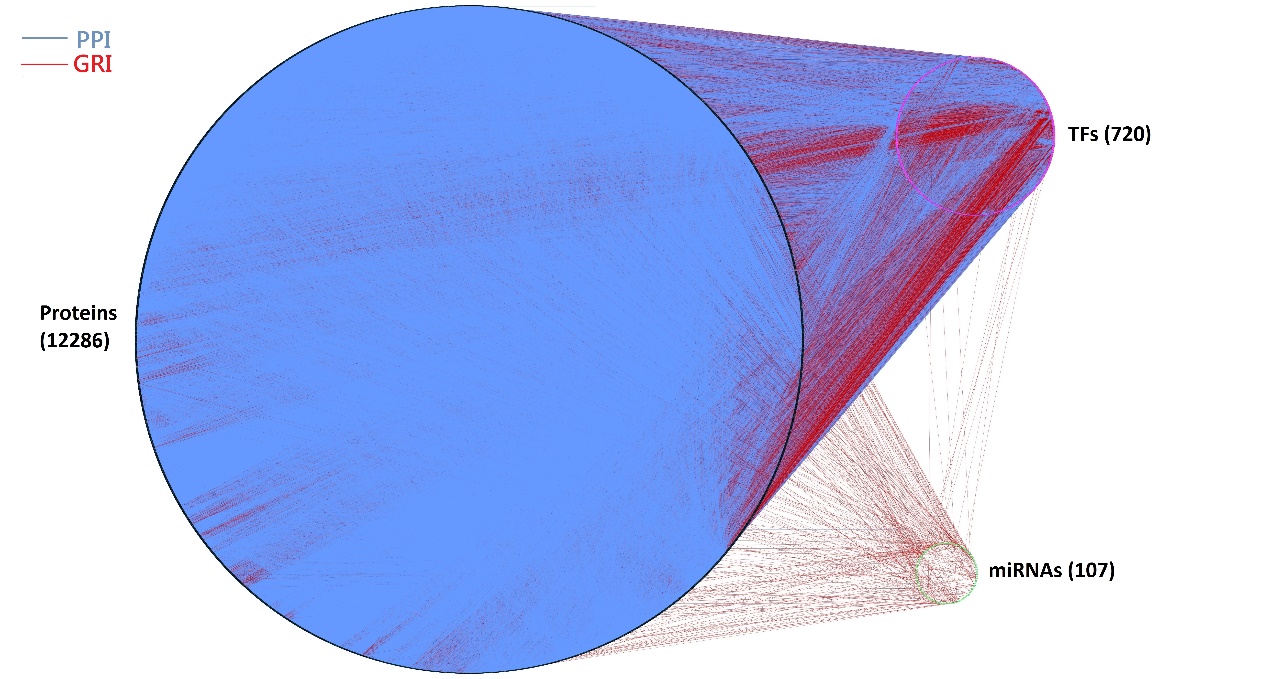


Figure S2. The genetic-and-epigenetic network (GEN) for NAFLD&NASH

The left circle represents the proteins (12286), the upper right circle represents the TFs (720) and the under right circle represents the miRNAs (107) in the GEN for NAFLD&NASH. Blue line represents protein-protein interaction (PPI) and red line represents gene regulatory information (GRI); PPIs are between protein circle and TF circle. GRIs are divided into the regulation of transcription factors on target genes and the regulation of miRNAs on target genes. The regulations of transcription factors on target genes are between TF circle and protein circle, and the regulations of miRNAs on target genes are between TF & protein circles and miRNA circle in the GEN for NAFLD&NASH.


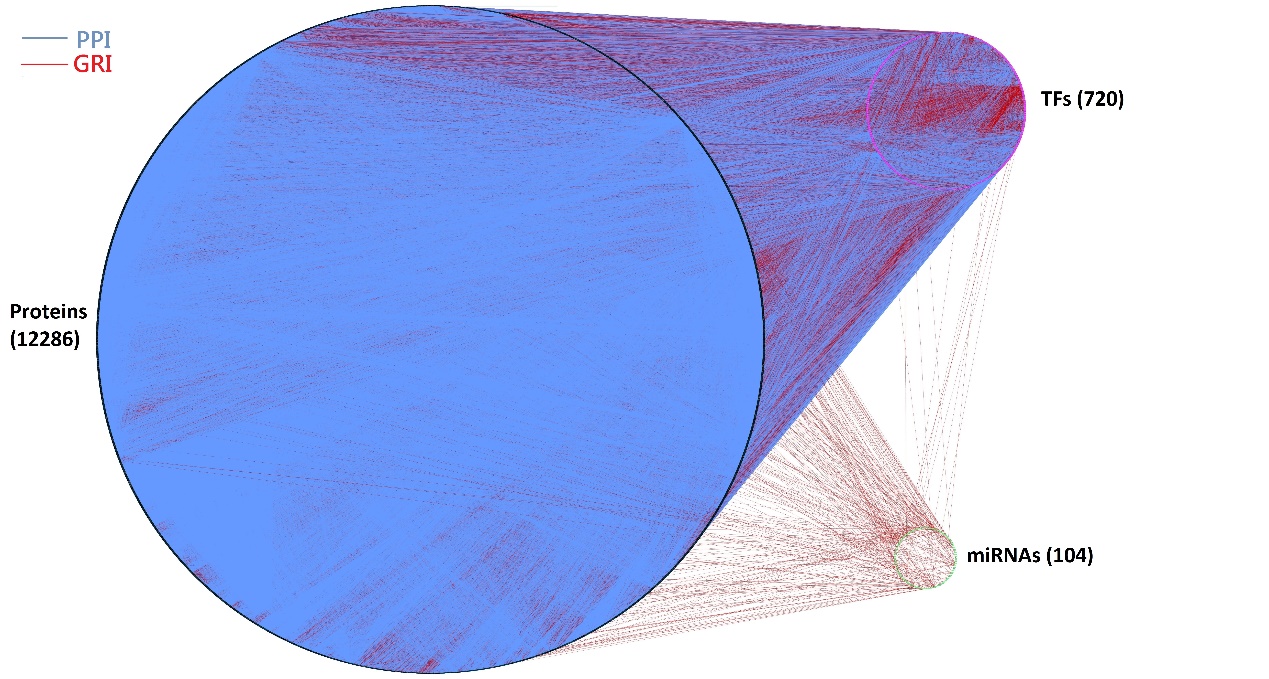


Figure S3. The genetic-and-epigenetic network (GEN) for PBC&PSC

The left circle represents the proteins (12286), the upper right circle represents the TFs (720) and the under right circle represents the miRNAs (104) in the GEN for PBC&PSC. Blue line represents protein-protein interaction (PPI) and red line represents gene regulatory information (GRI); PPIs are between protein circle and TF circle. GRIs are divided into the regulation of transcription factors on target genes and the regulation of miRNAs on target genes. The regulations of transcription factors on target genes are between TF circle and protein circle, and the regulations of miRNAs on target genes are between TF & protein circles and miRNA circle in the GEN for PBC&PSC.


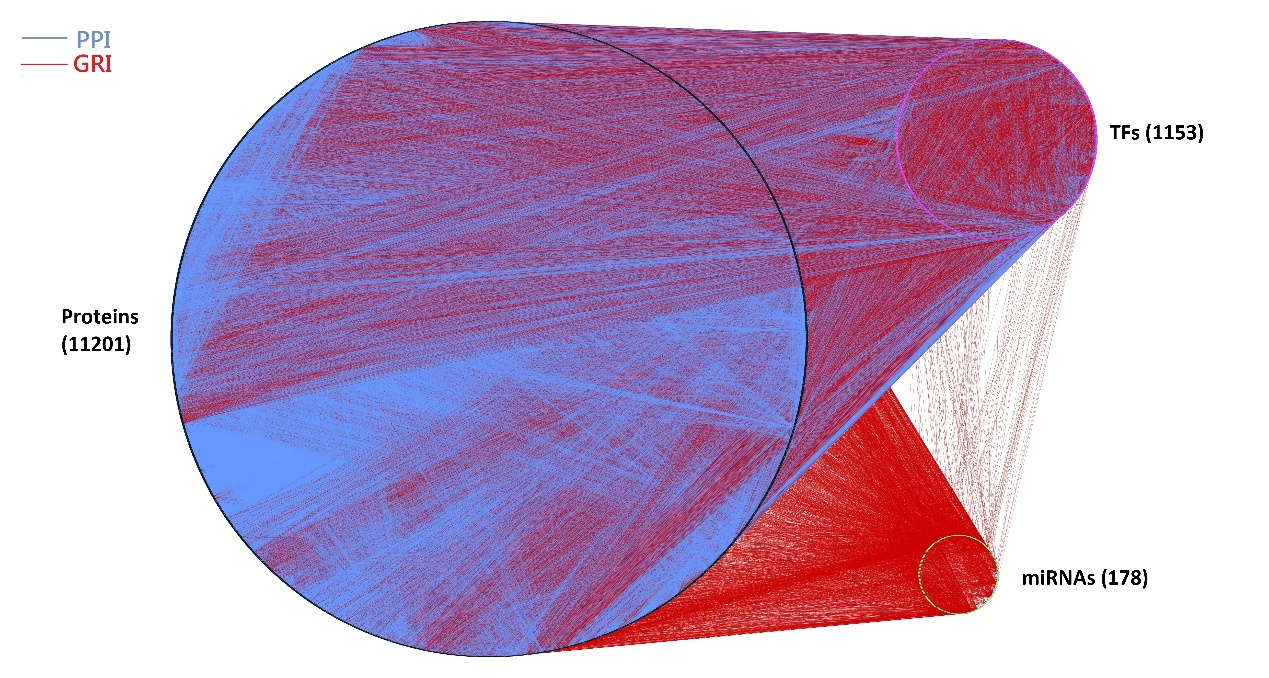


Figure S4. The genetic-and-epigenetic network (GEN) for HCC

The left circle represents the proteins (11201), the upper right circle represents the TFs (1153) and the under right circle represents the miRNAs (178) in the GEN for HCC. Blue line represents protein-protein interaction (PPI) and red line represents gene regulatory information (GRI); PPIs are between protein circle and TF circle. GRIs are divided into the regulation of transcription factors on target genes and the regulation of miRNAs on target genes. The regulations of transcriptions factors on target genes are between TF circle and protein circle, and the regulations of miRNAs on target genes are between TF & protein circles and miRNA circle in the GEN for HCC.


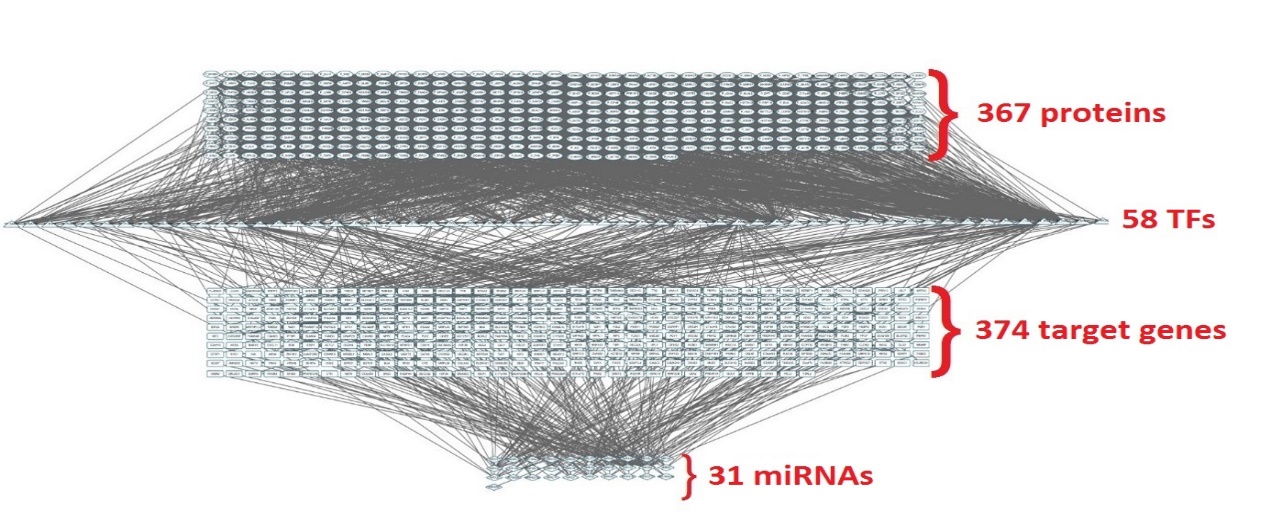


Figure S5. The core genetic-and-epigenetic network (GEN) for normal liver cell

The ellipse block represents protein, the triangle block represents transcription factor (TF), the rectangle block represents target gene and the diamond block represents miRNA. We extracted 5% proteins with top dependent score *D*(*k*) in (17) and their connecting genes and miRNAs from the real GEN for normal liver cell through principal network projection (PNP) to constitute core GEN for normal liver cell. As shown in the above figure, 3547 protein-protein interactions between proteins (367) and TFs (58), 299 regulations of transcription factors between TFs (58) and target genes (374), and 232 regulations of miRNAs between target genes (374) and miRNAs (31).


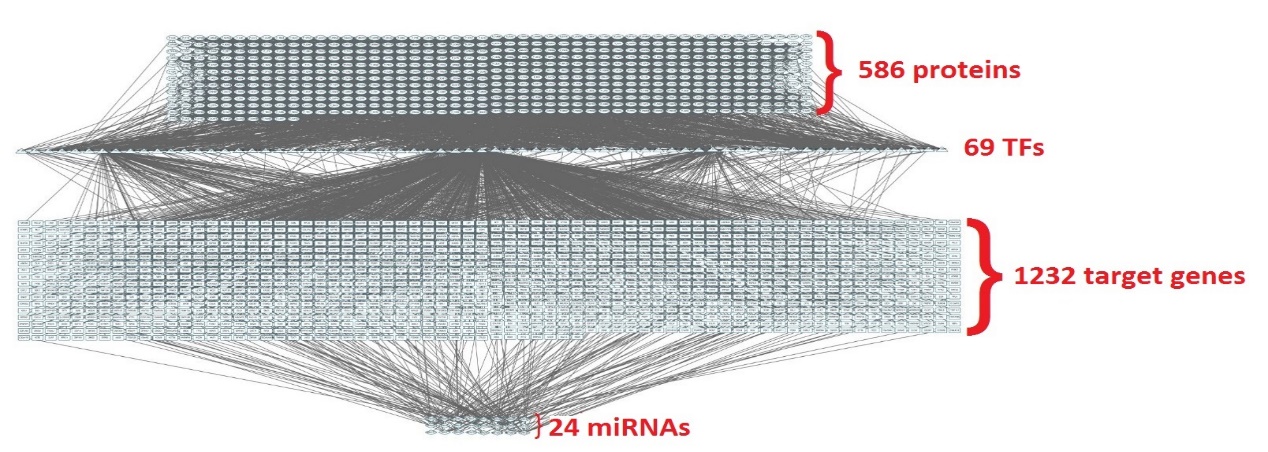


Figure S6. The core genetic-and-epigenetic network (GEN) for NAFLD&NASH

The ellipse block represents protein, the triangle block represents transcription factor (TF), the rectangle block represents target gene and the diamond block represents miRNA. We extracted 5% proteins with top dependent score *D*(*k*) in (17) and their connecting genes and miRNAs from the real GEN for NAFLD&NASH through principal network projection (PNP) to constitute core GEN for NAFLD&NASH. As shown in the above figure, 5271 protein-protein interactions between proteins (586) and TFs (69), 1163 regulations of transcription factors between TFs (69) and target genes (1232), and 197 regulations of miRNAs between target genes (1232) and miRNAs (24).


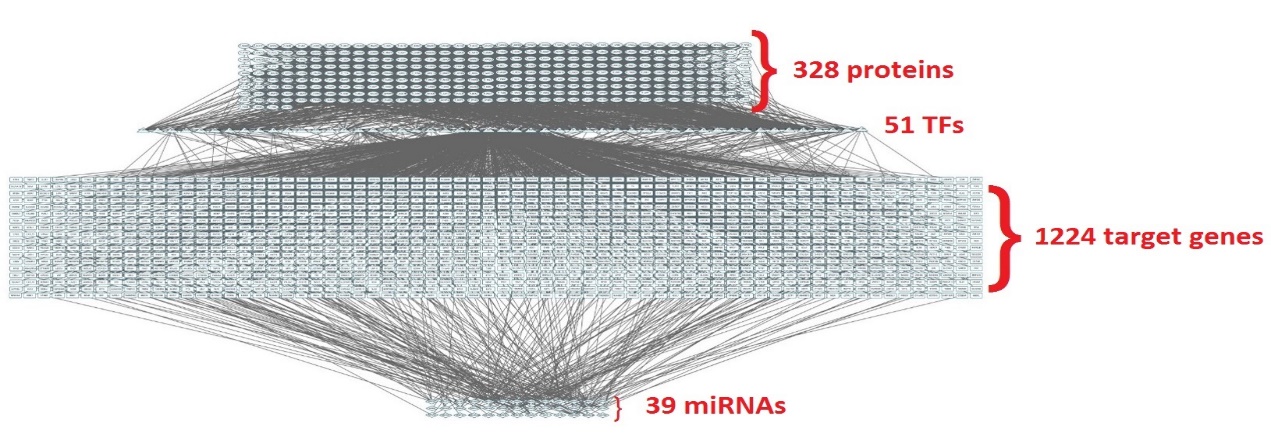


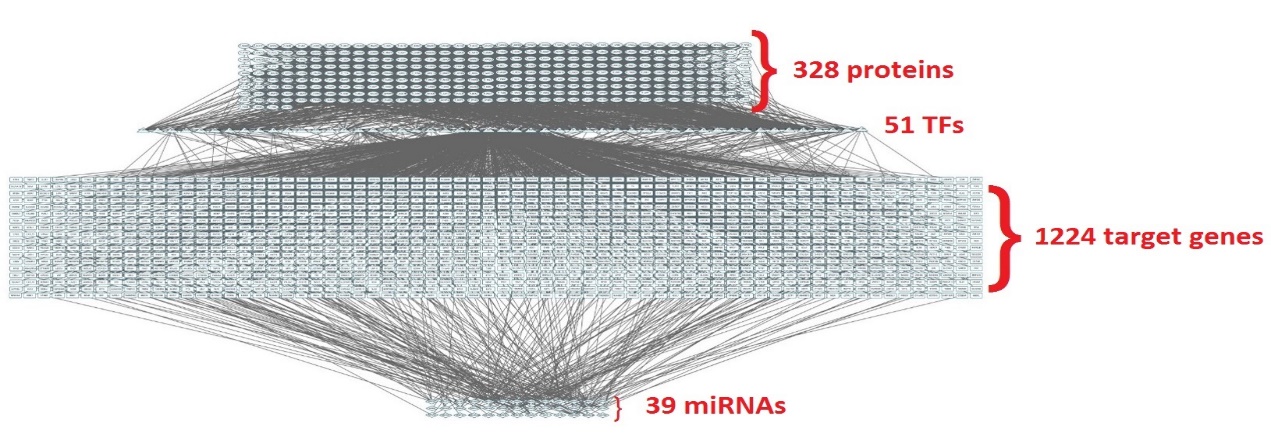


Figure S7. The core genetic-and-epigenetic network (GEN) for PBC&PSC

The ellipse block represents protein, the triangle block represents transcription factor (TF), the rectangle block represents target gene and the diamond block represents miRNA. We extracted 5% proteins with top dependent score *D*(*k*) in (17) and their connecting genes and miRNAs from the real GEN for PBC&PSC through principal network projection (PNP) to constitute core GEN for PBC&PSC. As shown in the above figure, 2384 protein-protein interactions between proteins (328) and TFs (51), 1061 regulations of transcription factors between TFs (51) and target genes (1224), and 263 regulations of miRNAs between target genes (1224) and miRNAs (39).


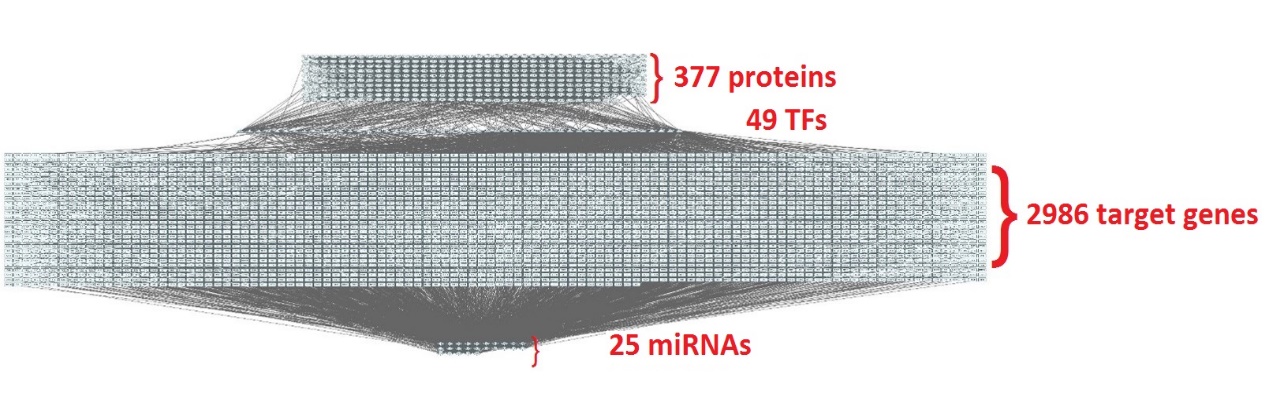


Figure S8. The core genetic-and-epigenetic network (GEN) for HCC

The ellipse block represents protein, the triangle block represents transcription factor (TF), the rectangle block represents target gene and the diamond block represents miRNA. We extracted 5% proteins with top dependent score *D*(*k*) in (17) and their connecting genes and miRNAs from the real GEN for HCC through principal network projection (PNP) to constitute core GEN for HCC. As shown in the above figure, 1571 protein-protein interactions between proteins (377) and TFs (49), 1241 regulations of transcription factors between TFs (49) and target genes (2986), and 2165 regulations of miRNAs between target genes (2986) and miRNAs (25).

Table S1. The projection values of the 37 core network biomarkers from normal liver cell to HCC through NAFLD&NASH in Figures 2­–3

37 proteins are selected from core GENs at three different liver diseases.

| PNP projection value *D*(*k*) of 37 core network biomarkers | | | |
| --- | --- | --- | --- |
| Core network biomarkers | **Normal liver cell** | **NAFLD&NASH** | **HCC** |
| ACTA2 | 2.08005^*^ | 0.20013^*^ | 0.00021^*^ |
| AIFM1 | 1090.292^*^ | 0.422186^*^ | 0.000109^*^ |
| APP | 0.45333 | 0.099171^*^ | 10.81104^*^ |
| CCDC8 | 62.45443^*^ | 0.046173^*^ | 0.000137^*^ |
| EGFR | 8.026025^*^ | 0.028813^*^ | 9.7E-05^*^ |
| GRB2 | 0.267095 | 0.043076^*^ | 0.000284^*^ |
| NEDD8 | 68.01829^*^ | 0.064802^*^ | 0.000102^*^ |
| SHC1 | 236.492^*^ | 0.066081^*^ | 0.001444^*^ |
| TUBA1C | 0.328937 | 0.036197^*^ | 6.7E-05^*^ |
| TUBB6 | 0.20169 | 0.157314^*^ | 5.56E-05^*^ |
| ALK | 0.272643 | 0.192363^*^ | 1.16E-09 |
| IL4R | 0.247634 | 0.100802^*^ | 1.99E-10 |
| GPR37 | 0.017353 | 0.05553^*^ | 6.83E-05^*^ |
| FRAT2 | 2.21E-05 | 5.16E-07^*^ | 1.66E-13 |
| HIST2H2BE | 0.505817^*^ | 0.000592^*^ | 2.7E-06 |
| HSPB1 | 0.205844 | 0.039457^*^ | 1.11E-05^*^ |
| ZNF480 | 0.015186 | 2.81E-08^*^ | 4.45E-13 |
| ALDOB | 0.005312 | 4.71E-06^*^ | 7.27E-06^*^ |
| ZYX | 0.047939 | 0.000107^*^ | 9.27E-07 |
| YBX1 | 0.006952 | 0.000354^*^ | 4.9E-06 |
| TP53 | 0.209012 | 0.025742^*^ | 4.76E-06 |
| STAT5A | 0.15274 | 0.000877^*^ | 3.03E-07 |
| AR | 0.005961 | 0.002358^*^ | 6.47E-09 |
| TELO2 | 0.005568 | 0.000884^*^ | 2.51E-11 |
| PXN | 0.050216 | 7.65E-05^*^ | 2.92E-06 |
| TBP | 0.024884 | 0.001792^*^ | 4.39E-08 |
| REPIN1 | 0.035269 | 1.15E-05^*^ | 1.36E-11 |
| HSF1 | 0.063824 | 0.018874^*^ | 4.54E-11 |
| GATA1 | 0.059625 | 0.027028^*^ | 5.86E-08 |
| ETS1 | 0.011066 | 1.12E-05^*^ | 9.8E-06^*^ |
| STUB1 | 0.124764 | 0.060566^*^ | 5.71E-06 |
| RFC5 | 0.16271 | 0.000112^*^ | 1.62E-08 |
| RPL30 | 0.005587 | 0.000376^*^ | 1.26E-06 |
| TRMT1 | 0.00026 | 8.76E-06^*^ | 2.08E-12 |
| GSK3B | 0.042492 | 0.003226^*^ | 3.7E-06 |
| UBC | 21.31796^*^ | 0.090865^*^ | 1.64E-06 |
| ALB | 0.003366 | 0.000131^*^ | 3673156^*^ |

^*^ indicates this protein is core protein in the core GEN of this liver condition.

Table S2. The projection values of the 40 core network biomarkers from normal liver cell to HCC through PBC&PSC in Figures 4­–5

40 proteins are selected from core GENs at three different liver diseases.

| PNP projection value *D*(*k*) of 40 core network biomarkers | | | |
| --- | --- | --- | --- |
| Core network biomarkers | **Normal liver cell** | **PBC&PSC** | **HCC** |
| ACTB | 0.613303^*^ | 1.081368^*^ | 0.000193^*^ |
| ADRB2 | 177.5427^*^ | 9.789294^*^ | 6.66E-05^*^ |
| APP | 0.45333 | 1.591937^*^ | 10.81104^*^ |
| CSNK2A1 | 0.26214 | 76.30621^*^ | 0.006644^*^ |
| EGFR | 8.026025^*^ | 11.1033^*^ | 9.7E-05^*^ |
| HUWE1 | 0.304621 | 1.107902^*^ | 0.000114^*^ |
| LMNB1 | 0.785651 | 1.711448^*^ | 6.61E-05 |
| PCK1 | 0.313342 | 2.58952^*^ | 0.00044^*^ |
| PPP2CA | 0.238486 | 36.76655^*^ | 0.000118^*^ |
| SMAD5 | 0.221115 | 124.6156^*^ | 6.18E-05^*^ |
| TSC22D1 | 0.434322 | 2.418161^*^ | 36.8852^*^ |
| ESR1 | 9.046137^*^ | 13.25506^*^ | 1.2E-05^*^ |
| PGR | 0.353102 | 2.47519^*^ | 7.77E-11 |
| RYR2 | 0.008007 | 39.77336^*^ | 644.8481^*^ |
| FRAT2 | 2.21E-05 | 0.014579^*^ | 1.66E-13 |
| HIST2H2BE | 0.505817^*^ | 13.23298^*^ | 2.7E-06 |
| ZNF480 | 0.015186 | 0.00042^*^ | 4.45E-13 |
| H3F3A | 0.077837 | 0.336896^*^ | 3.25E-11 |
| TIMP1 | 0.001577 | 0.023749^*^ | 2.87E-11 |
| ALDOB | 0.005312 | 0.034654^*^ | 7.27E-06^*^ |
| IGF2 | 0.016394 | 0.001712^*^ | 7.38E-10 |
| AR | 0.005961 | 0.145934^*^ | 6.47E-09 |
| TP53 | 0.209012 | 44.2936^*^ | 4.76E-06 |
| SRF | 0.122186 | 1.110673^*^ | 1.8E-10 |
| NRF1 | 8.7E-05 | 0.025152^*^ | 1.67E-09 |
| STAT5A | 0.15274 | 14.36718^*^ | 3.03E-07 |
| RPL23A | 0.085344 | 0.115819^*^ | 1.33E-05^*^ |
| SP3 | 0.017279 | 0.050799^*^ | 9.05E-11 |
| CEBPA | 0.037711 | 0.63064^*^ | 0.000139^*^ |
| BPTF | 0.033085 | 0.287476^*^ | 1.14E-07 |
| ETS1 | 0.011066 | 0.119931^*^ | 9.8E-06^*^ |
| MDC1 | 0.072077 | 0.24633^*^ | 7.14E-06 |
| RFC5 | 0.16271 | 0.265407^*^ | 1.62E-08 |
| RPL30 | 0.005587 | 0.179549^*^ | 1.26E-06 |
| TRMT1 | 0.00026 | 0.110787^*^ | 2.08E-12 |
| UBC | 21.31796^*^ | 101.8022^*^ | 1.64E-06 |
| GSK3B | 0.042492 | 18.13513^*^ | 3.7E-06 |
| Transferrin | 0.003288 | 0.01209^*^ | 2.28E-08 |
| CEBPE | 0.011566 | 0.215386^*^ | 2.08E-08 |
| GATA1 | 0.059625 | 34.9052^*^ | 5.86E-08 |

^*^ indicates this protein is core protein in the core GEN of this liver condition

Table S3. A list of ranked drugs to prevent hepatocarcinogenesis of NAFLD&NASH (A) and PBC&PSC (B) based on the drug response microarray data in cmap database.

| A | Drugs to prevent hepatocarcinogenesis of NAFLD&NASH | B | Drugs to prevent hepatocarcinogenesis of PBC&PSC |
| --- | --- | --- | --- |
|  | chlorpropamide |  | chlorpropamide |
|  | tolbutamide |  | tolbutamide |
|  | verapamil |  | verapamil |
|  | deferoxamine |  | deferoxamine |
|  | resveratrol |  | resveratrol |
|  | mesalazine |  | mesalazine |
|  | novobiocin |  | novobiocin |
|  | bucladesine |  | bucladesine |
|  | probucol |  | probucol |
|  | sulindac |  | sulindac |
|  | sulfasalazine |  | sulfasalazine |
|  | butirosin |  | butirosin |
|  | pentamidine |  | pentamidine |
|  | SC-19220 |  | SC-19220 |
|  | tetraethylenepentamine |  | tetraethylenepentamine |
|  | carbamazepine |  | carbamazepine |
|  | SB-202190 |  | SB-202190 |
|  | PHA-00846566E |  | PHA-00846566E |
|  | alsterpaullone |  | alsterpaullone |
|  | 0316684-0000 |  | 0316684-0000 |
|  | GW-8510 |  | GW-8510 |
|  | 0198306-0000 |  | 0198306-0000 |
|  | CAY-10397 |  | CAY-10397 |
|  | pyrvinium |  | pyrvinium |
|  | SB-203580 |  | SB-203580 |
|  | lomustine |  | lomustine |
|  | AR-A014418 |  | AR-A014418 |
|  | morantel |  | morantel |
|  | CP-863187 |  | CP-863187 |
|  | 4,5-dianilinophthalimide |  | 4,5-dianilinophthalimide |
|  | artemisinin |  | artemisinin |
|  | flunarizine |  | flunarizine |
|  | meteneprost |  | meteneprost |
|  | MG-262 |  | MG-262 |
|  | CP-944629 |  | CP-944629 |
|  | indometacin |  | indometacin |
|  | pergolide |  | pergolide |
|  | BAS-012416453 |  | BAS-012416453 |
|  | 0297417-0002B |  | 0297417-0002B |
|  | thapsigargin |  | thapsigargin |
|  | suramin sodium |  | suramin sodium |
|  | triprolidine |  | triprolidine |
|  | glafenine |  | glafenine |
|  | NS-398 |  | NS-398 |
|  | 16-phenyltetranorprostaglandin E2 |  | 16-phenyltetranorprostaglandin E2 |
|  | vinblastine |  | vinblastine |
|  | azathioprine |  | azathioprine |
|  | dyclonine |  | dyclonine |
|  | scriptaid |  | scriptaid |
|  | BCB000040 |  | BCB000040 |
|  | CP-645525-01 |  | CP-645525-01 |
|  | BCB000038 |  | BCB000038 |
|  | felodipine |  | felodipine |
|  | moxisylyte |  | moxisylyte |
|  | enalapril |  | enalapril |
|  | H-89 |  | H-89 |
|  | CP-319743 |  | CP-319743 |
|  | IC-86621 |  | IC-86621 |
|  | 11-deoxy-16,16-dimethylprostaglandin E2 |  | 11-deoxy-16,16-dimethylprostaglandin E2 |
|  | SC-58125 |  | SC-58125 |
|  | hydroflumethiazide |  | hydroflumethiazide |
|  | laudanosine |  | laudanosine |
|  | AG-012559 |  | AG-012559 |
|  | semustine |  | semustine |
|  | monensin |  | monensin |
|  | harmine |  | harmine |
|  | PHA-00816795 |  | PHA-00816795 |
|  | 15(S)-15-methylprostaglandin E2 |  | 15(S)-15-methylprostaglandin E2 |
|  | oxaprozin |  | oxaprozin |
|  | 1,4-chrysenequinone |  | 1,4-chrysenequinone |
|  | MS-275 |  | MS-275 |
|  | BCB000039 |  | BCB000039 |
|  | valproic acid |  | valproic acid |
|  | rofecoxib |  | rofecoxib |
|  | carmustine |  | carmustine |
|  | AH-6809 |  | AH-6809 |
|  | copper sulfate |  | copper sulfate |
|  | trazodone |  | trazodone |
|  | 0179445-0000 |  | 0179445-0000 |
|  | AH-23848 |  | AH-23848 |
|  | disopyramide |  | disopyramide |
|  | 8-azaguanine |  | 8-azaguanine |
|  | PHA-00851261E |  | PHA-00851261E |
|  | pirinixic acid |  | pirinixic acid |
|  | 6-bromoindirubin-3-oxime |  | 6-bromoindirubin-3-oxime |
|  | SC-560 |  | SC-560 |
|  | pyridoxine |  | pyridoxine |
|  | pimethixene |  | pimethixene |
|  | prednisolone |  | prednisolone |
|  | flufenamic acid |  | flufenamic acid |
|  | thiamphenicol |  | thiamphenicol |
|  | bromperidol |  | bromperidol |
|  | mesoridazine |  | mesoridazine |
|  | PHA-00745360 |  | PHA-00745360 |
|  | irinotecan |  | irinotecan |
|  | tacrolimus |  | tacrolimus |
|  | mercaptopurine |  | mercaptopurine |
|  | noretynodrel |  | noretynodrel |
|  | isoxicam |  | isoxicam |
|  | acetohexamide |  | acetohexamide |
|  | thalidomide |  | thalidomide |
|  | triflusal |  | triflusal |
|  | Prestwick-559 |  | Prestwick-559 |
|  | talampicillin |  | talampicillin |
|  | metformin |  | metformin |
|  | LM-1685 |  | LM-1685 |
|  | dexpanthenol |  | dexpanthenol |
|  | acetazolamide |  | acetazolamide |
|  | naproxen |  | naproxen |
|  | chloroquine |  | chloroquine |
|  | etamivan |  | etamivan |
|  | aminoglutethimide |  | aminoglutethimide |
|  | nifedipine |  | nifedipine |
|  | doxycycline |  | doxycycline |
|  | monocrotaline |  | monocrotaline |
|  | STOCK1N-28457 |  | STOCK1N-28457 |
|  | metrifonate |  | metrifonate |
|  | minocycline |  | minocycline |
|  | levocabastine |  | levocabastine |
|  | 0175029-0000 |  | 0175029-0000 |
|  | arachidonic acid |  | arachidonic acid |
|  | quercetin |  | quercetin |
|  | iloprost |  | iloprost |
|  | adiphenine |  | adiphenine |
|  | PNU-0230031 |  | PNU-0230031 |
|  | pioglitazone |  | pioglitazone |
|  | cobalt chloride |  | cobalt chloride |
|  | epirizole |  | epirizole |
|  | atracurium besilate |  | atracurium besilate |
|  | phenindione |  | phenindione |
|  | clemastine |  | clemastine |
|  | spaglumic acid |  | spaglumic acid |
|  | 0173570-0000 |  | 0173570-0000 |
|  | CP-690334-01 |  | CP-690334-01 |
|  | fulvestrant |  | fulvestrant |
|  | diclofenac |  | diclofenac |
|  | nitrendipine |  | nitrendipine |
|  | fisetin |  | fisetin |
|  | phentolamine |  | phentolamine |
|  | triflupromazine |  | triflupromazine |
|  | staurosporine |  | staurosporine |
|  | fasudil |  | fasudil |
|  | chlorpromazine |  | chlorpromazine |
|  | prochlorperazine |  | prochlorperazine |
|  | levamisole |  | levamisole |
|  | pargyline |  | pargyline |
|  | chlorphenesin |  | chlorphenesin |
|  | ethosuximide |  | ethosuximide |
|  | tomatidine |  | tomatidine |
|  | ipratropium bromide |  | ipratropium bromide |
|  | doxepin |  | doxepin |
|  | naftifine |  | naftifine |
|  | dexverapamil |  | dexverapamil |
|  | tretinoin |  | tretinoin |
|  | clofibrate |  | clofibrate |
|  | exisulind |  | exisulind |
|  | W-13 |  | W-13 |
|  | quinpirole |  | quinpirole |
|  | nalbuphine |  | nalbuphine |
|  | picotamide |  | picotamide |
|  | albendazole |  | albendazole |
|  | triamterene |  | triamterene |
|  | dihydroergocristine |  | dihydroergocristine |
|  | atropine |  | atropine |
|  | alprostadil |  | alprostadil |
|  | doxorubicin |  | doxorubicin |
|  | mecamylamine |  | mecamylamine |
|  | Prestwick-1082 |  | Prestwick-1082 |
|  | daunorubicin |  | daunorubicin |
|  | PF-00562151-00 |  | PF-00562151-00 |
|  | H-7 |  | H-7 |
|  | phenformin |  | phenformin |
|  | phenyl biguanide |  | phenyl biguanide |
|  | alpha-estradiol |  | alpha-estradiol |
|  | dexamethasone |  | dexamethasone |
|  | tamoxifen |  | tamoxifen |
|  | raloxifene |  | raloxifene |
|  | celecoxib |  | celecoxib |
|  | tomelukast |  | tomelukast |
|  | TTNPB |  | TTNPB |
|  | LY-294002 |  | LY-294002 |
|  | ciclosporin |  | ciclosporin |
|  | MK-886 |  | MK-886 |
|  | genistein |  | genistein |
|  | fludrocortisone |  | fludrocortisone |
|  | monastrol |  | monastrol |
|  | NU-1025 |  | NU-1025 |
|  | N-phenylanthranilic acid |  | N-phenylanthranilic acid |
|  | arachidonyltrifluoromethane |  | arachidonyltrifluoromethane |
|  | sodium phenylbutyrate |  | sodium phenylbutyrate |
|  | imatinib |  | imatinib |
|  | wortmannin |  | wortmannin |
|  | thioridazine |  | thioridazine |
|  | trifluoperazine |  | trifluoperazine |
|  | oligomycin |  | oligomycin |
|  | calmidazolium |  | calmidazolium |
|  | butein |  | butein |
|  | nocodazole |  | nocodazole |
|  | colchicine |  | colchicine |
|  | benserazide |  | benserazide |
|  | paclitaxel |  | paclitaxel |
|  | docosahexaenoic acid ethyl ester |  | docosahexaenoic acid ethyl ester |
|  | colforsin |  | colforsin |
|  | rottlerin |  | rottlerin |
|  | prazosin |  | prazosin |
|  | Y-27632 |  | Y-27632 |
|  | blebbistatin |  | blebbistatin |
|  | quinostatin |  | quinostatin |
|  | ikarugamycin |  | ikarugamycin |
|  | ionomycin |  | ionomycin |
|  | depudecin |  | depudecin |
|  | celastrol |  | celastrol |
|  | HC toxin |  | HC toxin |
|  | topiramate |  | topiramate |
|  | decitabine |  | decitabine |
|  | vorinostat |  | vorinostat |
|  | 3-hydroxy-DL-kynurenine |  | 3-hydroxy-DL-kynurenine |
|  | yohimbine |  | yohimbine |
|  | BW-B70C |  | BW-B70C |
|  | tyrphostin AG-1478 |  | tyrphostin AG-1478 |
|  | procainamide |  | procainamide |
|  | benfluorex |  | benfluorex |
|  | naftidrofuryl |  | naftidrofuryl |
|  | ketoconazole |  | ketoconazole |
|  | benzbromarone |  | benzbromarone |
|  | domperidone |  | domperidone |
|  | arecoline |  | arecoline |
|  | anisomycin |  | anisomycin |
|  | corticosterone |  | corticosterone |
|  | lactobionic acid |  | lactobionic acid |
|  | metrizamide |  | metrizamide |
|  | cefotetan |  | cefotetan |
|  | cefadroxil |  | cefadroxil |
|  | mebhydrolin |  | mebhydrolin |
|  | myricetin |  | myricetin |
|  | bacampicillin |  | bacampicillin |
|  | buflomedil |  | buflomedil |
|  | digitoxigenin |  | digitoxigenin |
|  | carteolol |  | carteolol |
|  | naringenin |  | naringenin |
|  | primaquine |  | primaquine |
|  | amantadine |  | amantadine |
|  | betulinic acid |  | betulinic acid |
|  | chlorogenic acid |  | chlorogenic acid |
|  | clindamycin |  | clindamycin |
|  | ampyrone |  | ampyrone |
|  | ranitidine |  | ranitidine |
|  | pentetrazol |  | pentetrazol |
|  | tiratricol |  | tiratricol |
|  | oxolinic acid |  | oxolinic acid |
|  | flumequine |  | flumequine |
|  | aztreonam |  | aztreonam |
|  | hydrastinine |  | hydrastinine |
|  | metampicillin |  | metampicillin |
|  | mafenide |  | mafenide |
|  | cimetidine |  | cimetidine |
|  | prednisone |  | prednisone |
|  | phenazone |  | phenazone |
|  | tolazoline |  | tolazoline |
|  | tolnaftate |  | tolnaftate |
|  | metronidazole |  | metronidazole |
|  | bromocriptine |  | bromocriptine |
|  | dehydrocholic acid |  | dehydrocholic acid |
|  | chlorhexidine |  | chlorhexidine |
|  | ampicillin |  | ampicillin |
|  | diltiazem |  | diltiazem |
|  | josamycin |  | josamycin |
|  | lynestrenol |  | lynestrenol |
|  | danazol |  | danazol |
|  | perphenazine |  | perphenazine |
|  | chlortetracycline |  | chlortetracycline |
|  | guanabenz |  | guanabenz |
|  | glibenclamide |  | glibenclamide |
|  | labetalol |  | labetalol |
|  | oxytetracycline |  | oxytetracycline |
|  | guanethidine |  | guanethidine |
|  | clonidine |  | clonidine |
|  | fipexide |  | fipexide |
|  | mepacrine |  | mepacrine |
|  | bupropion |  | bupropion |
|  | etacrynic acid |  | etacrynic acid |
|  | clomipramine |  | clomipramine |
|  | mepyramine |  | mepyramine |
|  | amodiaquine |  | amodiaquine |
|  | praziquantel |  | praziquantel |
|  | fendiline |  | fendiline |
|  | sulfinpyrazone |  | sulfinpyrazone |
|  | mebeverine |  | mebeverine |
|  | streptomycin |  | streptomycin |
|  | tremorine |  | tremorine |
|  | furosemide |  | furosemide |
|  | chlortalidone |  | chlortalidone |
|  | bambuterol |  | bambuterol |
|  | ketotifen |  | ketotifen |
|  | terbutaline |  | terbutaline |
|  | alfuzosin |  | alfuzosin |
|  | practolol |  | practolol |
|  | dobutamine |  | dobutamine |
|  | betaxolol |  | betaxolol |
|  | hemicholinium |  | hemicholinium |
|  | clopamide |  | clopamide |
|  | kanamycin |  | kanamycin |
|  | zaprinast |  | zaprinast |
|  | clenbuterol |  | clenbuterol |
|  | hycanthone |  | hycanthone |
|  | brinzolamide |  | brinzolamide |
|  | chlormezanone |  | chlormezanone |
|  | maprotiline |  | maprotiline |
|  | adenosine phosphate |  | adenosine phosphate |
|  | ambroxol |  | ambroxol |
|  | clofazimine |  | clofazimine |
|  | vanoxerine |  | vanoxerine |
|  | etoposide |  | etoposide |
|  | meticrane |  | meticrane |
|  | dapsone |  | dapsone |
|  | piracetam |  | piracetam |
|  | molsidomine |  | molsidomine |
|  | cyclizine |  | cyclizine |
|  | thiamine |  | thiamine |
|  | mometasone |  | mometasone |
|  | lobelanidine |  | lobelanidine |
|  | raubasine |  | raubasine |
|  | ajmaline |  | ajmaline |
|  | stachydrine |  | stachydrine |
|  | cytisine |  | cytisine |
|  | lobeline |  | lobeline |
|  | calycanthine |  | calycanthine |
|  | hydroquinine |  | hydroquinine |
|  | berberine |  | berberine |
|  | demecarium bromide |  | demecarium bromide |
|  | racecadotril |  | racecadotril |
|  | (+)-chelidonine |  | (+)-chelidonine |
|  | isocorydine |  | isocorydine |
|  | aceclofenac |  | aceclofenac |
|  | retrorsine |  | retrorsine |
|  | xylazine |  | xylazine |
|  | cinchonine |  | cinchonine |
|  | co-dergocrine mesilate |  | co-dergocrine mesilate |
|  | diflorasone |  | diflorasone |
|  | gramine |  | gramine |
|  | yohimbic acid |  | yohimbic acid |
|  | boldine |  | boldine |
|  | solanine |  | solanine |
|  | sulmazole |  | sulmazole |
|  | vitexin |  | vitexin |
|  | trihexyphenidyl |  | trihexyphenidyl |
|  | Prestwick-642 |  | Prestwick-642 |
|  | picrotoxinin |  | picrotoxinin |
|  | gelsemine |  | gelsemine |
|  | canadine |  | canadine |
|  | Prestwick-682 |  | Prestwick-682 |
|  | Prestwick-692 |  | Prestwick-692 |
|  | succinylsulfathiazole |  | succinylsulfathiazole |
|  | benzocaine |  | benzocaine |
|  | flunisolide |  | flunisolide |
|  | mepenzolate bromide |  | mepenzolate bromide |
|  | eldeline |  | eldeline |
|  | pempidine |  | pempidine |
|  | promazine |  | promazine |
|  | citiolone |  | citiolone |
|  | benfotiamine |  | benfotiamine |
|  | Prestwick-664 |  | Prestwick-664 |
|  | Prestwick-674 |  | Prestwick-674 |
|  | heliotrine |  | heliotrine |
|  | sulfamerazine |  | sulfamerazine |
|  | isosorbide |  | isosorbide |
|  | acacetin |  | acacetin |
|  | methylbenzethonium chloride |  | methylbenzethonium chloride |
|  | sulfachlorpyridazine |  | sulfachlorpyridazine |
|  | lanatoside C |  | lanatoside C |
|  | beta-escin |  | beta-escin |
|  | lycorine |  | lycorine |
|  | ethotoin |  | ethotoin |
|  | chlorcyclizine |  | chlorcyclizine |
|  | pramocaine |  | pramocaine |
|  | myosmine |  | myosmine |
|  | benzamil |  | benzamil |
|  | foliosidine |  | foliosidine |
|  | gossypol |  | gossypol |
|  | karakoline |  | karakoline |
|  | diphenylpyraline |  | diphenylpyraline |
|  | finasteride |  | finasteride |
|  | betonicine |  | betonicine |
|  | pentolonium |  | pentolonium |
|  | fenbufen |  | fenbufen |
|  | lomefloxacin |  | lomefloxacin |
|  | hydralazine |  | hydralazine |
|  | 6-benzylaminopurine |  | 6-benzylaminopurine |
|  | metoclopramide |  | metoclopramide |
|  | ketoprofen |  | ketoprofen |
|  | nefopam |  | nefopam |
|  | orphenadrine |  | orphenadrine |
|  | camptothecin |  | camptothecin |
|  | indapamide |  | indapamide |
|  | mexiletine |  | mexiletine |
|  | ethisterone |  | ethisterone |
|  | vincamine |  | vincamine |
|  | dantrolene |  | dantrolene |
|  | fenbendazole |  | fenbendazole |
|  | proglumide |  | proglumide |
|  | meclozine |  | meclozine |
|  | naringin |  | naringin |
|  | progesterone |  | progesterone |
|  | velnacrine |  | velnacrine |
|  | melatonin |  | melatonin |
|  | amphotericin B |  | amphotericin B |
|  | dicloxacillin |  | dicloxacillin |
|  | dinoprost |  | dinoprost |
|  | metixene |  | metixene |
|  | vigabatrin |  | vigabatrin |
|  | pirenperone |  | pirenperone |
|  | ciclopirox |  | ciclopirox |
|  | hexetidine |  | hexetidine |
|  | cefotiam |  | cefotiam |
|  | nitrofural |  | nitrofural |
|  | biperiden |  | biperiden |
|  | iohexol |  | iohexol |
|  | thiostrepton |  | thiostrepton |
|  | selegiline |  | selegiline |
|  | L-methionine sulfoximine |  | L-methionine sulfoximine |
|  | cefaclor |  | cefaclor |
|  | trimethadione |  | trimethadione |
|  | rifampicin |  | rifampicin |
|  | methoxamine |  | methoxamine |
|  | nifuroxazide |  | nifuroxazide |
|  | tiaprofenic acid |  | tiaprofenic acid |
|  | sisomicin |  | sisomicin |
|  | lovastatin |  | lovastatin |
|  | ethionamide |  | ethionamide |
|  | R-atenolol |  | R-atenolol |
|  | pregnenolone |  | pregnenolone |
|  | vancomycin |  | vancomycin |
|  | nystatin |  | nystatin |
|  | tenoxicam |  | tenoxicam |
|  | suxibuzone |  | suxibuzone |
|  | skimmianine |  | skimmianine |
|  | ricinine |  | ricinine |
|  | estropipate |  | estropipate |
|  | tetryzoline |  | tetryzoline |
|  | fluorometholone |  | fluorometholone |
|  | etanidazole |  | etanidazole |
|  | anabasine |  | anabasine |
|  | hexestrol |  | hexestrol |
|  | trioxysalen |  | trioxysalen |
|  | cefalotin |  | cefalotin |
|  | fluorocurarine |  | fluorocurarine |
|  | cefmetazole |  | cefmetazole |
|  | strophanthidin |  | strophanthidin |
|  | cefuroxime |  | cefuroxime |
|  | altizide |  | altizide |
|  | sulfaquinoxaline |  | sulfaquinoxaline |
|  | nadide |  | nadide |
|  | terazosin |  | terazosin |
|  | butamben |  | butamben |
|  | alclometasone |  | alclometasone |
|  | ramifenazone |  | ramifenazone |
|  | streptozocin |  | streptozocin |
|  | sulfamethizole |  | sulfamethizole |
|  | phenazopyridine |  | phenazopyridine |
|  | sulfapyridine |  | sulfapyridine |
|  | leflunomide |  | leflunomide |
|  | podophyllotoxin |  | podophyllotoxin |
|  | propidium iodide |  | propidium iodide |
|  | pheneticillin |  | pheneticillin |
|  | metoprolol |  | metoprolol |
|  | medrysone |  | medrysone |
|  | bendroflumethiazide |  | bendroflumethiazide |
|  | eucatropine |  | eucatropine |
|  | flecainide |  | flecainide |
|  | ethoxyquin |  | ethoxyquin |
|  | sulfadimidine |  | sulfadimidine |
|  | dicoumarol |  | dicoumarol |
|  | diethylstilbestrol |  | diethylstilbestrol |
|  | tinidazole |  | tinidazole |
|  | guaifenesin |  | guaifenesin |
|  | lithocholic acid |  | lithocholic acid |
|  | alpha-ergocryptine |  | alpha-ergocryptine |
|  | atractyloside |  | atractyloside |
|  | merbromin |  | merbromin |
|  | sulfadimethoxine |  | sulfadimethoxine |
|  | hexylcaine |  | hexylcaine |
|  | ebselen |  | ebselen |
|  | mevalolactone |  | mevalolactone |
|  | cicloheximide |  | cicloheximide |
|  | amrinone |  | amrinone |
|  | azlocillin |  | azlocillin |
|  | butacaine |  | butacaine |
|  | methazolamide |  | methazolamide |
|  | clidinium bromide |  | clidinium bromide |
|  | iopromide |  | iopromide |
|  | sulfamonomethoxine |  | sulfamonomethoxine |
|  | corynanthine |  | corynanthine |
|  | harmalol |  | harmalol |
|  | Prestwick-984 |  | Prestwick-984 |
|  | cyclopenthiazide |  | cyclopenthiazide |
|  | etofenamate |  | etofenamate |
|  | apramycin |  | apramycin |
|  | zalcitabine |  | zalcitabine |
|  | zuclopenthixol |  | zuclopenthixol |
|  | gabexate |  | gabexate |
|  | benzathine benzylpenicillin |  | benzathine benzylpenicillin |
|  | ozagrel |  | ozagrel |
|  | tribenoside |  | tribenoside |
|  | phensuximide |  | phensuximide |
|  | cyclic adenosine monophosphate |  | cyclic adenosine monophosphate |
|  | loracarbef |  | loracarbef |
|  | procarbazine |  | procarbazine |
|  | bethanechol |  | bethanechol |
|  | levopropoxyphene |  | levopropoxyphene |
|  | nafcillin |  | nafcillin |
|  | liothyronine |  | liothyronine |
|  | etidronic acid |  | etidronic acid |
|  | theophylline |  | theophylline |
|  | spectinomycin |  | spectinomycin |
|  | cefsulodin |  | cefsulodin |
|  | benzthiazide |  | benzthiazide |
|  | procyclidine |  | procyclidine |
|  | Prestwick-864 |  | Prestwick-864 |
|  | piromidic acid |  | piromidic acid |
|  | tetroquinone |  | tetroquinone |
|  | amiprilose |  | amiprilose |
|  | simvastatin |  | simvastatin |
|  | trimipramine |  | trimipramine |
|  | suprofen |  | suprofen |
|  | oxolamine |  | oxolamine |
|  | indoprofen |  | indoprofen |
|  | tolmetin |  | tolmetin |
|  | arcaine |  | arcaine |
|  | chloropyramine |  | chloropyramine |
|  | (+/-)-catechin |  | (+/-)-catechin |
|  | propantheline bromide |  | propantheline bromide |
|  | carbenoxolone |  | carbenoxolone |
|  | levobunolol |  | levobunolol |
|  | Prestwick-857 |  | Prestwick-857 |
|  | paromomycin |  | paromomycin |
|  | scopolamine |  | scopolamine |
|  | furazolidone |  | furazolidone |
|  | nadolol |  | nadolol |
|  | lasalocid |  | lasalocid |
|  | iocetamic acid |  | iocetamic acid |
|  | iodixanol |  | iodixanol |
|  | paracetamol |  | paracetamol |
|  | ioversol |  | ioversol |
|  | diclofenamide |  | diclofenamide |
|  | latamoxef |  | latamoxef |
|  | dimethadione |  | dimethadione |
|  | ganciclovir |  | ganciclovir |
|  | phthalylsulfathiazole |  | phthalylsulfathiazole |
|  | capsaicin |  | capsaicin |
|  | Prestwick-860 |  | Prestwick-860 |
|  | 3-acetylcoumarin |  | 3-acetylcoumarin |
|  | hymecromone |  | hymecromone |
|  | (+)-isoprenaline |  | (+)-isoprenaline |
|  | nizatidine |  | nizatidine |
|  | pentetic acid |  | pentetic acid |
|  | crotamiton |  | crotamiton |
|  | bemegride |  | bemegride |
|  | esculin |  | esculin |
|  | caffeic acid |  | caffeic acid |
|  | thioperamide |  | thioperamide |
|  | eticlopride |  | eticlopride |
|  | bretylium tosilate |  | bretylium tosilate |
|  | propranolol |  | propranolol |
|  | digoxigenin |  | digoxigenin |
|  | felbinac |  | felbinac |
|  | diloxanide |  | diloxanide |
|  | 2-aminobenzenesulfonamide |  | 2-aminobenzenesulfonamide |
|  | xamoterol |  | xamoterol |
|  | primidone |  | primidone |
|  | pralidoxime |  | pralidoxime |
|  | (-)-atenolol |  | (-)-atenolol |
|  | meglumine |  | meglumine |
|  | butyl hydroxybenzoate |  | butyl hydroxybenzoate |
|  | metyrapone |  | metyrapone |
|  | estrone |  | estrone |
|  | tyloxapol |  | tyloxapol |
|  | tonzonium bromide |  | tonzonium bromide |
|  | (-)-MK-801 |  | (-)-MK-801 |
|  | clioquinol |  | clioquinol |
|  | fluspirilene |  | fluspirilene |
|  | altretamine |  | altretamine |
|  | megestrol |  | megestrol |
|  | oxybenzone |  | oxybenzone |
|  | pipemidic acid |  | pipemidic acid |
|  | dexibuprofen |  | dexibuprofen |
|  | flurbiprofen |  | flurbiprofen |
|  | prasterone |  | prasterone |
|  | dioxybenzone |  | dioxybenzone |
|  | etynodiol |  | etynodiol |
|  | nimodipine |  | nimodipine |
|  | nilutamide |  | nilutamide |
|  | chrysin |  | chrysin |
|  | adrenosterone |  | adrenosterone |
|  | nabumetone |  | nabumetone |
|  | bacitracin |  | bacitracin |
|  | adipiodone |  | adipiodone |
|  | octopamine |  | octopamine |
|  | atropine methonitrate |  | atropine methonitrate |
|  | nisoxetine |  | nisoxetine |
|  | aminocaproic acid |  | aminocaproic acid |
|  | denatonium benzoate |  | denatonium benzoate |
|  | flucloxacillin |  | flucloxacillin |
|  | isradipine |  | isradipine |
|  | halofantrine |  | halofantrine |
|  | rilmenidine |  | rilmenidine |
|  | pizotifen |  | pizotifen |
|  | alfaxalone |  | alfaxalone |
|  | articaine |  | articaine |
|  | enilconazole |  | enilconazole |
|  | ribavirin |  | ribavirin |
|  | deptropine |  | deptropine |
|  | isometheptene |  | isometheptene |
|  | clofilium tosylate |  | clofilium tosylate |
|  | quinidine |  | quinidine |
|  | lidoflazine |  | lidoflazine |
|  | mitoxantrone |  | mitoxantrone |
|  | hydrocortisone |  | hydrocortisone |
|  | methoxsalen |  | methoxsalen |
|  | azacitidine |  | azacitidine |
|  | levomepromazine |  | levomepromazine |
|  | dienestrol |  | dienestrol |
|  | cromoglicic acid |  | cromoglicic acid |
|  | ifosfamide |  | ifosfamide |
|  | imidurea |  | imidurea |
|  | prednicarbate |  | prednicarbate |
|  | meprylcaine |  | meprylcaine |
|  | cyproterone |  | cyproterone |
|  | saquinavir |  | saquinavir |
|  | Prestwick-1085 |  | Prestwick-1085 |
|  | cyclopentolate |  | cyclopentolate |
|  | methanthelinium bromide |  | methanthelinium bromide |
|  | ciprofibrate |  | ciprofibrate |
|  | dorzolamide |  | dorzolamide |
|  | piretanide |  | piretanide |
|  | tropine |  | tropine |
|  | chloropyrazine |  | chloropyrazine |
|  | (-)-isoprenaline |  | (-)-isoprenaline |
|  | ondansetron |  | ondansetron |
|  | thiethylperazine |  | thiethylperazine |
|  | benzylpenicillin |  | benzylpenicillin |
|  | mephenytoin |  | mephenytoin |
|  | cefepime |  | cefepime |
|  | oxyphenbutazone |  | oxyphenbutazone |
|  | PNU-0251126 |  | PNU-0251126 |
|  | 0317956-0000 |  | 0317956-0000 |
|  | chlorambucil |  | chlorambucil |
|  | levcycloserine |  | levcycloserine |
|  | nialamide |  | nialamide |
|  | quinethazone |  | quinethazone |
|  | santonin |  | santonin |
|  | Prestwick-1080 |  | Prestwick-1080 |
|  | homosalate |  | homosalate |
|  | Prestwick-1100 |  | Prestwick-1100 |
|  | parbendazole |  | parbendazole |
|  | ciclacillin |  | ciclacillin |
|  | 2,6-dimethylpiperidine |  | 2,6-dimethylpiperidine |
|  | spiradoline |  | spiradoline |
|  | withaferin A |  | withaferin A |
|  | citalopram |  | citalopram |
|  | paroxetine |  | paroxetine |
|  | CP-320650-01 |  | CP-320650-01 |
|  | rifabutin |  | rifabutin |
|  | clobetasol |  | clobetasol |
|  | timolol |  | timolol |
|  | nomegestrol |  | nomegestrol |
|  | AG-013608 |  | AG-013608 |
|  | valinomycin |  | valinomycin |
|  | rotenone |  | rotenone |
|  | PF-00875133-00 |  | PF-00875133-00 |
|  | PF-00539745-00 |  | PF-00539745-00 |
|  | PF-01378883-00 |  | PF-01378883-00 |
|  | erastin |  | erastin |
|  | 3-nitropropionic acid |  | 3-nitropropionic acid |
|  | valdecoxib |  | valdecoxib |
|  | PF-00539758-00 |  | PF-00539758-00 |
|  | STOCK1N-35215 |  | STOCK1N-35215 |
|  | orlistat |  | orlistat |
|  | 0225151-0000 |  | 0225151-0000 |
|  | C-75 |  | C-75 |
|  | F0447-0125 |  | F0447-0125 |
|  | dinoprostone |  | dinoprostone |
|  | amitriptyline |  | amitriptyline |
|  | nordihydroguaiaretic acid |  | nordihydroguaiaretic acid |
|  | acetylsalicylic acid |  | acetylsalicylic acid |
|  | sirolimus |  | sirolimus |
|  | trichostatin A |  | trichostatin A |
|  | troglitazone |  | troglitazone |
|  | fluphenazine |  | fluphenazine |
|  | dopamine |  | dopamine |
|  | gefitinib |  | gefitinib |
|  | 1,5-isoquinolinediol |  | 1,5-isoquinolinediol |
|  | dimethyloxalylglycine |  | dimethyloxalylglycine |
|  | pararosaniline |  | pararosaniline |
|  | clotrimazole |  | clotrimazole |
|  | alvespimycin |  | alvespimycin |
|  | demecolcine |  | demecolcine |
|  | 12,13-EODE |  | 12,13-EODE |
|  | tyrphostin AG-825 |  | tyrphostin AG-825 |
|  | phenanthridinone |  | phenanthridinone |
|  | DL-PPMP |  | DL-PPMP |
|  | MG-132 |  | MG-132 |
|  | thioguanosine |  | thioguanosine |
|  | amoxicillin |  | amoxicillin |
|  | bepridil |  | bepridil |
|  | guanfacine |  | guanfacine |
|  | ritodrine |  | ritodrine |
|  | droperidol |  | droperidol |
|  | clebopride |  | clebopride |
|  | androsterone |  | androsterone |
|  | suloctidil |  | suloctidil |
|  | coralyne |  | coralyne |
|  | carcinine |  | carcinine |
|  | N-acetylmuramic acid |  | N-acetylmuramic acid |
|  | cyclobenzaprine |  | cyclobenzaprine |
|  | atropine oxide |  | atropine oxide |
|  | thioproperazine |  | thioproperazine |
|  | triamcinolone |  | triamcinolone |
|  | isoniazid |  | isoniazid |
|  | tranexamic acid |  | tranexamic acid |
|  | piroxicam |  | piroxicam |
|  | etodolac |  | etodolac |
|  | chlorzoxazone |  | chlorzoxazone |
|  | tranylcypromine |  | tranylcypromine |
|  | hyoscyamine |  | hyoscyamine |
|  | pentoxifylline |  | pentoxifylline |
|  | dicycloverine |  | dicycloverine |
|  | amylocaine |  | amylocaine |
|  | cotinine |  | cotinine |
|  | hesperetin |  | hesperetin |
|  | oxybutynin |  | oxybutynin |
|  | spiperone |  | spiperone |
|  | mifepristone |  | mifepristone |
|  | diperodon |  | diperodon |
|  | betamethasone |  | betamethasone |
|  | nicardipine |  | nicardipine |
|  | clorgiline |  | clorgiline |
|  | ascorbic acid |  | ascorbic acid |
|  | metitepine |  | metitepine |
|  | cefoperazone |  | cefoperazone |
|  | procaine |  | procaine |
|  | todralazine |  | todralazine |
|  | diprophylline |  | diprophylline |
|  | sulfacetamide |  | sulfacetamide |
|  | dirithromycin |  | dirithromycin |
|  | imipenem |  | imipenem |
|  | prenylamine |  | prenylamine |
|  | tetrahydroalstonine |  | tetrahydroalstonine |
|  | piperlongumine |  | piperlongumine |
|  | pseudopelletierine |  | pseudopelletierine |
|  | isoflupredone |  | isoflupredone |
|  | canrenoic acid |  | canrenoic acid |
|  | glimepiride |  | glimepiride |
|  | dydrogesterone |  | dydrogesterone |
|  | famprofazone |  | famprofazone |
|  | fludroxycortide |  | fludroxycortide |
|  | helveticoside |  | helveticoside |
|  | griseofulvin |  | griseofulvin |
|  | riluzole |  | riluzole |
|  | nalidixic acid |  | nalidixic acid |
|  | mebendazole |  | mebendazole |
|  | ofloxacin |  | ofloxacin |
|  | tropicamide |  | tropicamide |
|  | flutamide |  | flutamide |
|  | fenofibrate |  | fenofibrate |
|  | perhexiline |  | perhexiline |
|  | digoxin |  | digoxin |
|  | neostigmine bromide |  | neostigmine bromide |
|  | bisacodyl |  | bisacodyl |
|  | colecalciferol |  | colecalciferol |
|  | carbimazole |  | carbimazole |
|  | menadione |  | menadione |
|  | niridazole |  | niridazole |
|  | ceforanide |  | ceforanide |
|  | serotonin |  | serotonin |
|  | 6-azathymine |  | 6-azathymine |
|  | atovaquone |  | atovaquone |
|  | tolazamide |  | tolazamide |
|  | tetramisole |  | tetramisole |
|  | colistin |  | colistin |
|  | benzethonium chloride |  | benzethonium chloride |
|  | kinetin |  | kinetin |
|  | delsoline |  | delsoline |
|  | abamectin |  | abamectin |
|  | napelline |  | napelline |
|  | demeclocycline |  | demeclocycline |
|  | levonorgestrel |  | levonorgestrel |
|  | cloperastine |  | cloperastine |
|  | fenoprofen |  | fenoprofen |
|  | furaltadone |  | furaltadone |
|  | spiramycin |  | spiramycin |
|  | isocarboxazid |  | isocarboxazid |
|  | thiamazole |  | thiamazole |
|  | alexidine |  | alexidine |
|  | calcium folinate |  | calcium folinate |
|  | mimosine |  | mimosine |
|  | sulfanilamide |  | sulfanilamide |
|  | sulfametoxydiazine |  | sulfametoxydiazine |
|  | drofenine |  | drofenine |
|  | pyrithyldione |  | pyrithyldione |
|  | alpha-yohimbine |  | alpha-yohimbine |
|  | salsolinol |  | salsolinol |
|  | Prestwick-691 |  | Prestwick-691 |
|  | mycophenolic acid |  | mycophenolic acid |
|  | metacycline |  | metacycline |
|  | naftopidil |  | naftopidil |
|  | etamsylate |  | etamsylate |
|  | tracazolate |  | tracazolate |
|  | proguanil |  | proguanil |
|  | risperidone |  | risperidone |
|  | piribedil |  | piribedil |
|  | etomidate |  | etomidate |
|  | S-propranolol |  | S-propranolol |
|  | tridihexethyl |  | tridihexethyl |
|  | ioxaglic acid |  | ioxaglic acid |
|  | penbutolol |  | penbutolol |
|  | viomycin |  | viomycin |
|  | roxithromycin |  | roxithromycin |
|  | theobromine |  | theobromine |
|  | doxazosin |  | doxazosin |
|  | aminophylline |  | aminophylline |
|  | profenamine |  | profenamine |
|  | equilin |  | equilin |
|  | propofol |  | propofol |
|  | monobenzone |  | monobenzone |
|  | rolipram |  | rolipram |
|  | aminohippuric acid |  | aminohippuric acid |
|  | lorglumide |  | lorglumide |
|  | terguride |  | terguride |
|  | florfenicol |  | florfenicol |
|  | N-acetyl-L-leucine |  | N-acetyl-L-leucine |
|  | idazoxan |  | idazoxan |
|  | bephenium hydroxynaphthoate |  | bephenium hydroxynaphthoate |
|  | desoxycortone |  | desoxycortone |
|  | promethazine |  | promethazine |
|  | proxymetacaine |  | proxymetacaine |
|  | protriptyline |  | protriptyline |
|  | remoxipride |  | remoxipride |
|  | gliquidone |  | gliquidone |
|  | Prestwick-972 |  | Prestwick-972 |
|  | tiletamine |  | tiletamine |
|  | pirlindole |  | pirlindole |
|  | Prestwick-983 |  | Prestwick-983 |
|  | sertaconazole |  | sertaconazole |
|  | piperidolate |  | piperidolate |
|  | dexpropranolol |  | dexpropranolol |
|  | verteporfin |  | verteporfin |
|  | piperacetazine |  | piperacetazine |
|  | propoxycaine |  | propoxycaine |
|  | phenoxybenzamine |  | phenoxybenzamine |
|  | PHA-00767505E |  | PHA-00767505E |
|  | estradiol |  | estradiol |
|  | exemestane |  | exemestane |
|  | 15-delta prostaglandin J2 |  | 15-delta prostaglandin J2 |
|  | tanespimycin |  | tanespimycin |
|  | sulindac sulfide |  | sulindac sulfide |
|  | monorden |  | monorden |
|  | 2-deoxy-D-glucose |  | 2-deoxy-D-glucose |
|  | rosiglitazone |  | rosiglitazone |
|  | clozapine |  | clozapine |
|  | haloperidol |  | haloperidol |
|  | oxamic acid |  | oxamic acid |
|  | HNMPA-(AM)3 |  | HNMPA-(AM)3 |
|  | 3-aminobenzamide |  | 3-aminobenzamide |
|  | geldanamycin |  | geldanamycin |
|  | 5666823 |  | 5666823 |
|  | tioguanine |  | tioguanine |
|  | splitomicin |  | splitomicin |
|  | U0125 |  | U0125 |
|  | 5252917 |  | 5252917 |
|  | 5255229 |  | 5255229 |
|  | 5211181 |  | 5211181 |
|  | 5248896 |  | 5248896 |
|  | 5224221 |  | 5224221 |
|  | 5279552 |  | 5279552 |
|  | 5253409 |  | 5253409 |
|  | 5230742 |  | 5230742 |
|  | 5182598 |  | 5182598 |
|  | 5186223 |  | 5186223 |
|  | 5286656 |  | 5286656 |
|  | 5149715 |  | 5149715 |
|  | 5162773 |  | 5162773 |
|  | 5152487 |  | 5152487 |
|  | 5213008 |  | 5213008 |
|  | 5186324 |  | 5186324 |
|  | 5114445 |  | 5114445 |
|  | 5151277 |  | 5151277 |
|  | 5109870 |  | 5109870 |
|  | 5140203 |  | 5140203 |
|  | 5252917 |  | 5252917 |
|  | 5255229 |  | 5255229 |
|  | 5211181 |  | 5211181 |
|  | 5248896 |  | 5248896 |
|  | 5224221 |  | 5224221 |
|  | 5279552 |  | 5279552 |
|  | 5253409 |  | 5253409 |
|  | 5230742 |  | 5230742 |
|  | 5182598 |  | 5182598 |
|  | (-)-catechin |  | (-)-catechin |
|  | cytochalasin B |  | cytochalasin B |
|  | pentoxyverine |  | pentoxyverine |
|  | clomifene |  | clomifene |
|  | zoxazolamine |  | zoxazolamine |
|  | N6-methyladenosine |  | N6-methyladenosine |
|  | chlorprothixene |  | chlorprothixene |
|  | cefalexin |  | cefalexin |
|  | bezafibrate |  | bezafibrate |
|  | dequalinium chloride |  | dequalinium chloride |
|  | oxantel |  | oxantel |
|  | tacrine |  | tacrine |
|  | dextromethorphan |  | dextromethorphan |
|  | buspirone |  | buspirone |
|  | bisoprolol |  | bisoprolol |
|  | flupentixol |  | flupentixol |
|  | fusidic acid |  | fusidic acid |
|  | hesperidin |  | hesperidin |
|  | testosterone |  | testosterone |
|  | ouabain |  | ouabain |
|  | fusaric acid |  | fusaric acid |
|  | cefixime |  | cefixime |
|  | calcium pantothenate |  | calcium pantothenate |
|  | levothyroxine sodium |  | levothyroxine sodium |
|  | carbarsone |  | carbarsone |
|  | carisoprodol |  | carisoprodol |
|  | cyanocobalamin |  | cyanocobalamin |
|  | SR-95531 |  | SR-95531 |
|  | lumicolchicine |  | lumicolchicine |
|  | dl-alpha tocopherol |  | dl-alpha tocopherol |
|  | apigenin |  | apigenin |
|  | 7-aminocephalosporanic acid |  | 7-aminocephalosporanic acid |
|  | ginkgolide A |  | ginkgolide A |
|  | lysergol |  | lysergol |
|  | piperine |  | piperine |
|  | pepstatin |  | pepstatin |
|  | N-acetyl-L-aspartic acid |  | N-acetyl-L-aspartic acid |
|  | Chicago Sky Blue 6B |  | Chicago Sky Blue 6B |
|  | brompheniramine |  | brompheniramine |
|  | SR-95639A |  | SR-95639A |
|  | meclocycline |  | meclocycline |
|  | naltrexone |  | naltrexone |
|  | mefloquine |  | mefloquine |
|  | astemizole |  | astemizole |
|  | ivermectin |  | ivermectin |
|  | diazoxide |  | diazoxide |
|  | disulfiram |  | disulfiram |
|  | chlorphenamine |  | chlorphenamine |
|  | isoconazole |  | isoconazole |
|  | nicergoline |  | nicergoline |
|  | gallamine triethiodide |  | gallamine triethiodide |
|  | aminophenazone |  | aminophenazone |
|  | acetylsalicylsalicylic acid |  | acetylsalicylsalicylic acid |
|  | nomifensine |  | nomifensine |
|  | spironolactone |  | spironolactone |
|  | terfenadine |  | terfenadine |
|  | neomycin |  | neomycin |
|  | mianserin |  | mianserin |
|  | dizocilpine |  | dizocilpine |
|  | pirenzepine |  | pirenzepine |
|  | cefotaxime |  | cefotaxime |
|  | dihydrostreptomycin |  | dihydrostreptomycin |
|  | pindolol |  | pindolol |
|  | acenocoumarol |  | acenocoumarol |
|  | tetracycline |  | tetracycline |
|  | dihydroergotamine |  | dihydroergotamine |
|  | midodrine |  | midodrine |
|  | norfloxacin |  | norfloxacin |
|  | etofylline |  | etofylline |
|  | pyrantel |  | pyrantel |
|  | antimycin A |  | antimycin A |
|  | scopolamine N-oxide |  | scopolamine N-oxide |
|  | fenspiride |  | fenspiride |
|  | xylometazoline |  | xylometazoline |
|  | ornidazole |  | ornidazole |
|  | alverine |  | alverine |
|  | nimesulide |  | nimesulide |
|  | gemfibrozil |  | gemfibrozil |
|  | oxymetazoline |  | oxymetazoline |
|  | tolfenamic acid |  | tolfenamic acid |
|  | mefexamide |  | mefexamide |
|  | nifenazone |  | nifenazone |
|  | iproniazid |  | iproniazid |
|  | cloxacillin |  | cloxacillin |
|  | meclofenamic acid |  | meclofenamic acid |
|  | sulfathiazole |  | sulfathiazole |
|  | troleandomycin |  | troleandomycin |
|  | naphazoline |  | naphazoline |
|  | sulpiride |  | sulpiride |
|  | minaprine |  | minaprine |
|  | cinchocaine |  | cinchocaine |
|  | amiloride |  | amiloride |
|  | levodopa |  | levodopa |
|  | doxylamine |  | doxylamine |
|  | pyrimethamine |  | pyrimethamine |
|  | ticlopidine |  | ticlopidine |
|  | oxybuprocaine |  | oxybuprocaine |
|  | miconazole |  | miconazole |
|  | amprolium |  | amprolium |
|  | idoxuridine |  | idoxuridine |
|  | ethambutol |  | ethambutol |
|  | hexamethonium bromide |  | hexamethonium bromide |
|  | oxetacaine |  | oxetacaine |
|  | isoxsuprine |  | isoxsuprine |
|  | hydrochlorothiazide |  | hydrochlorothiazide |
|  | captopril |  | captopril |
|  | diflunisal |  | diflunisal |
|  | pheniramine |  | pheniramine |
|  | acebutolol |  | acebutolol |
|  | diphemanil metilsulfate |  | diphemanil metilsulfate |
|  | sulfaguanidine |  | sulfaguanidine |
|  | minoxidil |  | minoxidil |
|  | 4-hydroxyphenazone |  | 4-hydroxyphenazone |
|  | niclosamide |  | niclosamide |
|  | lidocaine |  | lidocaine |
|  | trimethobenzamide |  | trimethobenzamide |
|  | khellin |  | khellin |
|  | apomorphine |  | apomorphine |
|  | naloxone |  | naloxone |
|  | glipizide |  | glipizide |
|  | erythromycin |  | erythromycin |
|  | zimeldine |  | zimeldine |
|  | amoxapine |  | amoxapine |
|  | metolazone |  | metolazone |
|  | metanephrine |  | metanephrine |
|  | loxapine |  | loxapine |
|  | dipyridamole |  | dipyridamole |
|  | oleandomycin |  | oleandomycin |
|  | edrophonium chloride |  | edrophonium chloride |
|  | azacyclonol |  | azacyclonol |
|  | cyproheptadine |  | cyproheptadine |
|  | ciprofloxacin |  | ciprofloxacin |
|  | hydroxyzine |  | hydroxyzine |
|  | midecamycin |  | midecamycin |
|  | moroxydine |  | moroxydine |
|  | famotidine |  | famotidine |
|  | loperamide |  | loperamide |
|  | baclofen |  | baclofen |
|  | aciclovir |  | aciclovir |
|  | lisuride |  | lisuride |
|  | benzydamine |  | benzydamine |
|  | antazoline |  | antazoline |
|  | vinpocetine |  | vinpocetine |
|  | cinnarizine |  | cinnarizine |
|  | pimozide |  | pimozide |
|  | methylprednisolone |  | methylprednisolone |
|  | alprenolol |  | alprenolol |
|  | methapyrilene |  | methapyrilene |
|  | debrisoquine |  | debrisoquine |
|  | ketanserin |  | ketanserin |
|  | zidovudine |  | zidovudine |
|  | desipramine |  | desipramine |
|  | enoxacin |  | enoxacin |
|  | methotrexate |  | methotrexate |
|  | phenylpropanolamine |  | phenylpropanolamine |
|  | sulfafurazole |  | sulfafurazole |
|  | metergoline |  | metergoline |
|  | methylergometrine |  | methylergometrine |
|  | amikacin |  | amikacin |
|  | methyldopa |  | methyldopa |
|  | sulfaphenazole |  | sulfaphenazole |
|  | allantoin |  | allantoin |
|  | benzonatate |  | benzonatate |
|  | homatropine |  | homatropine |
|  | imipramine |  | imipramine |
|  | sulfadiazine |  | sulfadiazine |
|  | betazole |  | betazole |
|  | mefenamic acid |  | mefenamic acid |
|  | heptaminol |  | heptaminol |
|  | diphenhydramine |  | diphenhydramine |
|  | dosulepin |  | dosulepin |
|  | budesonide |  | budesonide |
|  | gliclazide |  | gliclazide |
|  | ceftazidime |  | ceftazidime |
|  | propafenone |  | propafenone |
|  | thiocolchicoside |  | thiocolchicoside |
|  | trimetazidine |  | trimetazidine |
|  | iobenguane |  | iobenguane |
|  | Trolox C |  | Trolox C |
|  | clorsulon |  | clorsulon |
|  | parthenolide |  | parthenolide |
|  | tubocurarine chloride |  | tubocurarine chloride |
|  | tetracaine |  | tetracaine |
|  | hydrastine hydrochloride |  | hydrastine hydrochloride |
|  | scoulerine |  | scoulerine |
|  | 10-methoxyharmalan |  | 10-methoxyharmalan |
|  | harmol |  | harmol |
|  | dipivefrine |  | dipivefrine |
|  | noscapine |  | noscapine |
|  | papaverine |  | papaverine |
|  | syrosingopine |  | syrosingopine |
|  | dacarbazine |  | dacarbazine |
|  | ellipticine |  | ellipticine |
|  | riboflavin |  | riboflavin |
|  | hydrocotarnine |  | hydrocotarnine |
|  | physostigmine |  | physostigmine |
|  | acepromazine |  | acepromazine |
|  | convolamine |  | convolamine |
|  | cinchonidine |  | cinchonidine |
|  | epivincamine |  | epivincamine |
|  | aconitine |  | aconitine |
|  | vinburnine |  | vinburnine |
|  | quipazine |  | quipazine |
|  | folic acid |  | folic acid |
|  | chloramphenicol |  | chloramphenicol |
|  | ursolic acid |  | ursolic acid |
|  | gentamicin |  | gentamicin |
|  | methocarbamol |  | methocarbamol |
|  | rescinnamine |  | rescinnamine |
|  | galantamine |  | galantamine |
|  | sparteine |  | sparteine |
|  | conessine |  | conessine |
|  | palmatine |  | palmatine |
|  | bicuculline |  | bicuculline |
|  | seneciphylline |  | seneciphylline |
|  | canavanine |  | canavanine |
|  | protoveratrine A |  | protoveratrine A |
|  | emetine |  | emetine |
|  | trimethylcolchicinic acid |  | trimethylcolchicinic acid |
|  | harmaline |  | harmaline |
|  | harman |  | harman |
|  | quinisocaine |  | quinisocaine |
|  | hydroxyachillin |  | hydroxyachillin |
|  | sulfabenzamide |  | sulfabenzamide |
|  | solasodine |  | solasodine |
|  | metamizole sodium |  | metamizole sodium |
|  | bromopride |  | bromopride |
|  | halcinonide |  | halcinonide |
|  | Prestwick-665 |  | Prestwick-665 |
|  | Prestwick-675 |  | Prestwick-675 |
|  | Prestwick-685 |  | Prestwick-685 |
|  | etiocholanolone |  | etiocholanolone |
|  | tiapride |  | tiapride |
|  | dilazep |  | dilazep |
|  | sulfamethoxazole |  | sulfamethoxazole |
|  | metaraminol |  | metaraminol |
|  | kawain |  | kawain |
|  | clemizole |  | clemizole |
|  | nitrofurantoin |  | nitrofurantoin |
|  | mephenesin |  | mephenesin |
|  | salbutamol |  | salbutamol |
|  | trimethoprim |  | trimethoprim |
|  | prilocaine |  | prilocaine |
|  | phenelzine |  | phenelzine |
|  | lisinopril |  | lisinopril |
|  | ifenprodil |  | ifenprodil |
|  | flavoxate |  | flavoxate |
|  | difenidol |  | difenidol |
|  | fenoterol |  | fenoterol |
|  | lincomycin |  | lincomycin |
|  | bufexamac |  | bufexamac |
|  | norethisterone |  | norethisterone |
|  | cortisone |  | cortisone |
|  | homochlorcyclizine |  | homochlorcyclizine |
|  | telenzepine |  | telenzepine |
|  | nortriptyline |  | nortriptyline |
|  | diethylcarbamazine |  | diethylcarbamazine |
|  | econazole |  | econazole |
|  | dropropizine |  | dropropizine |
|  | niflumic acid |  | niflumic acid |
|  | dimenhydrinate |  | dimenhydrinate |
|  | chenodeoxycholic acid |  | chenodeoxycholic acid |
|  | bupivacaine |  | bupivacaine |
|  | pinacidil |  | pinacidil |
|  | isotretinoin |  | isotretinoin |
|  | bumetanide |  | bumetanide |
|  | acemetacin |  | acemetacin |
|  | butoconazole |  | butoconazole |
|  | biotin |  | biotin |
|  | cephaeline |  | cephaeline |
|  | amiodarone |  | amiodarone |
|  | pilocarpine |  | pilocarpine |
|  | ergocalciferol |  | ergocalciferol |
|  | cisapride |  | cisapride |
|  | epiandrosterone |  | epiandrosterone |
|  | puromycin |  | puromycin |
|  | fluoxetine |  | fluoxetine |
|  | glycocholic acid |  | glycocholic acid |
|  | salsolidin |  | salsolidin |
|  | probenecid |  | probenecid |
|  | omeprazole |  | omeprazole |
|  | cetirizine |  | cetirizine |
|  | norcyclobenzaprine |  | norcyclobenzaprine |
|  | phenacetin |  | phenacetin |
|  | betahistine |  | betahistine |
|  | benperidol |  | benperidol |
|  | propylthiouracil |  | propylthiouracil |
|  | etifenin |  | etifenin |
|  | pyrazinamide |  | pyrazinamide |
|  | tiabendazole |  | tiabendazole |
|  | tobramycin |  | tobramycin |
|  | terconazole |  | terconazole |
|  | orciprenaline |  | orciprenaline |
|  | Prestwick-689 |  | Prestwick-689 |
|  | tetrandrine |  | tetrandrine |
|  | meclofenoxate |  | meclofenoxate |
|  | sulfamethoxypyridazine |  | sulfamethoxypyridazine |
|  | flumetasone |  | flumetasone |
|  | flunixin |  | flunixin |
|  | mephentermine |  | mephentermine |
|  | cefazolin |  | cefazolin |
|  | glycopyrronium bromide |  | glycopyrronium bromide |
|  | guanadrel |  | guanadrel |
|  | ribostamycin |  | ribostamycin |
|  | vidarabine |  | vidarabine |
|  | proadifen |  | proadifen |
|  | corbadrine |  | corbadrine |
|  | isoetarine |  | isoetarine |
|  | zomepirac |  | zomepirac |
|  | pridinol |  | pridinol |
|  | hecogenin |  | hecogenin |
|  | pipenzolate bromide |  | pipenzolate bromide |
|  | isopropamide iodide |  | isopropamide iodide |
|  | cinoxacin |  | cinoxacin |
|  | carbinoxamine |  | carbinoxamine |
|  | bergenin |  | bergenin |
|  | securinine |  | securinine |
|  | cefapirin |  | cefapirin |
|  | gabapentin |  | gabapentin |
|  | iopamidol |  | iopamidol |
|  | cefoxitin |  | cefoxitin |
|  | alimemazine |  | alimemazine |
|  | DL-thiorphan |  | DL-thiorphan |
|  | pronetalol |  | pronetalol |
|  | meptazinol |  | meptazinol |
|  | nifurtimox |  | nifurtimox |
|  | pancuronium bromide |  | pancuronium bromide |
|  | gibberellic acid |  | gibberellic acid |
|  | sitosterol |  | sitosterol |
|  | fluvoxamine |  | fluvoxamine |
|  | letrozole |  | letrozole |
|  | molindone |  | molindone |
|  | sotalol |  | sotalol |
|  | proscillaridin |  | proscillaridin |
|  | cefalonium |  | cefalonium |
|  | epitiostanol |  | epitiostanol |
|  | moxonidine |  | moxonidine |
|  | oxamniquine |  | oxamniquine |
|  | Prestwick-967 |  | Prestwick-967 |
|  | zardaverine |  | zardaverine |
|  | sanguinarine |  | sanguinarine |
|  | fluticasone |  | fluticasone |
|  | fursultiamine |  | fursultiamine |
|  | etilefrine |  | etilefrine |
|  | tocainide |  | tocainide |
|  | decamethonium bromide |  | decamethonium bromide |
|  | memantine |  | memantine |
|  | harpagoside |  | harpagoside |
|  | methyldopate |  | methyldopate |
|  | 3-acetamidocoumarin |  | 3-acetamidocoumarin |
|  | asiaticoside |  | asiaticoside |
|  | pivampicillin |  | pivampicillin |
|  | roxarsone |  | roxarsone |
|  | betulin |  | betulin |
|  | lymecycline |  | lymecycline |
|  | rimexolone |  | rimexolone |
|  | torasemide |  | torasemide |
|  | moracizine |  | moracizine |
|  | netilmicin |  | netilmicin |
|  | iopanoic acid |  | iopanoic acid |
|  | lansoprazole |  | lansoprazole |
|  | pivmecillinam |  | pivmecillinam |
|  | Prestwick-1083 |  | Prestwick-1083 |
|  | Prestwick-1103 |  | Prestwick-1103 |
|  | fosfosal |  | fosfosal |
|  | trichlormethiazide |  | trichlormethiazide |
|  | beclometasone |  | beclometasone |
|  | reserpine |  | reserpine |
|  | mestranol |  | mestranol |
|  | rolitetracycline |  | rolitetracycline |
|  | fluvastatin |  | fluvastatin |
|  | sulconazole |  | sulconazole |
|  | ethaverine |  | ethaverine |
|  | luteolin |  | luteolin |
|  | carbachol |  | carbachol |
|  | nicotinic acid |  | nicotinic acid |
|  | flucytosine |  | flucytosine |
|  | cantharidin |  | cantharidin |
|  | urapidil |  | urapidil |
|  | ursodeoxycholic acid |  | ursodeoxycholic acid |
|  | ketorolac |  | ketorolac |
|  | proxyphylline |  | proxyphylline |
|  | Prestwick-920 |  | Prestwick-920 |
|  | esculetin |  | esculetin |
|  | nipecotic acid |  | nipecotic acid |
|  | Prestwick-981 |  | Prestwick-981 |
|  | alfadolone |  | alfadolone |
|  | scopoletin |  | scopoletin |
|  | trapidil |  | trapidil |
|  | azapropazone |  | azapropazone |
|  | fluocinonide |  | fluocinonide |
|  | piperacillin |  | piperacillin |
|  | cefamandole |  | cefamandole |
|  | methacholine chloride |  | methacholine chloride |
|  | Prestwick-1084 |  | Prestwick-1084 |
|  | sulfadoxine |  | sulfadoxine |
|  | natamycin |  | natamycin |
|  | milrinone |  | milrinone |
|  | ronidazole |  | ronidazole |
|  | repaglinide |  | repaglinide |
|  | trifluridine |  | trifluridine |
|  | cycloserine |  | cycloserine |
|  | estriol |  | estriol |
|  | meropenem |  | meropenem |
|  | oxprenolol |  | oxprenolol |
|  | ramipril |  | ramipril |
|  | azaperone |  | azaperone |
|  | oxedrine |  | oxedrine |
|  | kaempferol |  | kaempferol |
|  | alcuronium chloride |  | alcuronium chloride |
|  | ticarcillin |  | ticarcillin |
|  | 5707885 |  | 5707885 |
|  | 5707885 |  | 5707885 |
|  | 5707885 |  | 5707885 |
|  | 5707885 |  | 5707885 |
|  | 5155877 |  | 5155877 |
|  | 5155877 |  | 5155877 |
|  | 5194442 |  | 5194442 |
|  | AG-028671 |  | AG-028671 |
|  | 5194442 |  | 5194442 |
|  | Gly-His-Lys |  | Gly-His-Lys |
|  | STOCK1N-35874 |  | STOCK1N-35874 |
|  | 16,16-dimethylprostaglandin E2 |  | 16,16-dimethylprostaglandin E2 |
|  | PNU-0293363 |  | PNU-0293363 |
|  | STOCK1N-35696 |  | STOCK1N-35696 |
|  | 5155877 |  | 5155877 |
|  | 5155877 |  | 5155877 |
|  | PHA-00665752 |  | PHA-00665752 |
|  | 5194442 |  | 5194442 |
|  | 5194442 |  | 5194442 |
